# *Saccharomyces boulardii* CNCM I-745 mitigates antibiotic-induced gut microbiome functional alterations independently of the host

**DOI:** 10.1080/19490976.2025.2575924

**Published:** 2025-11-07

**Authors:** Zhan Huang, Loic Brot, Rand Fatouh, Marius Bredon, Laura Creusot, Antoine Lefèvre, Antonin Lamazière, Jérémie H. Lefevre, Patrick Emond, Julien Planchais, Xavier Roux, Harry Sokol, Nathalie Rolhion

**Affiliations:** a Sorbonne Université, Inserm, Centre de Recherche Saint-Antoine, CRSA, Paris, France; b Gut, Liver & Microbiome Research (GLIMMER) FHU, Paris, France; c INSERM, Imaging Brain & Neuropsychiatry iBraiN U1253, Université de Tours, Tours, France; d Plateforme de Métabolomique et d'Analyses Chimiques, US61 ASB, Université de Tours, CHRU Tours, Inserm, Tours, France; e MetaboHUB-Tours, Tours, France; f Department of Clinical Metabolomics, AP-HP, Hôpital Saint Antoine, Sorbonne Université, Paris, France; g Department of Digestive Surgery, AP-HP, Hôpital Saint Antoine, Sorbonne Université, Paris, France; h Research and Development Center, Biocodex, Compiègne, France; i Department of Gastroenterology, AP-HP, Hôpital Saint Antoine, Sorbonne Université, Paris, France; j INRAE, UMR1319 Micalis, AgroParisTech, Jouy-en-Josas, France

**Keywords:** *Saccharomyces boulardii* CNCM I-745, antibiotic, gut microbiome, quantitative microbiota profiling, metabolomics, immune response

## Abstract

The probiotic *Saccharomyces boulardii* CNCM I-745 (Sb) is widely prescribed to alleviate antibiotic-induced diarrhea, yet its mode of action, particularly its potential direct effects on the gut microbiome, remains incompletely defined. This study aimed to evaluate whether Sb can directly mitigate antibiotic-induced gut microbiota dysbiosis and influence downstream host immune response. Using both static (MiPro) and dynamic (SHIME^®^) *in vitro* gut microbiota models, we assessed the effects of Sb supplementation under antibiotic treatment with amoxicillin/clavulanic acid (AMC) or vancomycin (Van). Quantitative microbiome profiling integrated with targeted metabolomics showed that Sb helped stabilize bacterial biomass, partially preserved metabolic functions, and restored the production of immunoregulatory metabolites propionate and indole-3-propionic acid under AMC treatment. In addition, *ex vivo* exposure of primary human immune cells (PBMCs) and intestinal mucosal tissue to microbiota modulated by Sb led to a significant reduction in pro-inflammatory cytokine secretion compared to microbiota not supplemented with Sb. Collectively, these results support a beneficial role for *S. boulardii* CNCM I-745 in preserving directly gut microbiome function and supporting host immune homeostasis during antibiotic treatment, particularly under AMC exposure. Our findings advance the understanding of probiotic-antibiotic-gut microbiome interactions, thereby guiding future optimization of microbiome-targeted adjuvant therapies.

## Introduction

Antibiotics play a pivotal role in the treatment of bacterial infections.^1^ However, advancements in the understanding of the human gut microbiome—now recognized as a “microbial organ” with significant roles in health and disease^2^—have prompted a reevaluation of antibiotic use. Antibiotic exposure disrupts the taxonomic composition and metabolic activity of resident intestinal microbiota^3^ and induces the expansion of antibiotic-resistant bacteria,^4^ which are potential triggers for developing chronic diseases later in life.^5^

To mitigate antibiotic-induced dysbiosis, probiotics have been clinically used as supplements.^6^ Among these, *Saccharomyces boulardii* CNCM I-745 (Sb) is one of the most studied and the most widely prescribed, particularly for the prevention of antibiotic-associated diarrhea.^7^ Numerous clinical studies have demonstrated that the use of Sb improves antibiotic-associated clinical outcomes.^8-11^ However, the mode of action of Sb has not been fully elucidated. While several trials have reported modest effects of Sb on microbiota composition, such as attenuating shifts in specific taxa,^12-14^ there is limited evidence that it substantially alters global microbiota diversity or structure. Moreover, the functional consequences of Sb administration on microbiome activity and metabolic output remain largely unexplored. Most prior studies have not employed multi-omics techniques, including metagenomics or metabolomics, to characterize Sb-associated changes in microbiota function. As a result, it remains unclear whether the clinical benefits of Sb stem from functionally relevant stabilization of microbiome activity during antibiotic stress.

Since Sb has well-documented direct effects on host epithelial and immune cells^15^^,^^16^ *in vivo* studies cannot disentangle whether observed microbiome changes are driven directly by Sb-gut microbiota interactions or indirectly through host-mediated feedback. *In vitro* microbiota models offer a critical opportunity to evaluate potential direct effects of Sb on the human microbiome under controlled, host-free conditions. These models retain donor-specific microbiota complexity and drug responsiveness,^17-20^ making them suitable for probing functional and compositional microbiome dynamics. A systematic investigation of the direct effects of Sb on the antibiotic-perturbed gut microbiome is essential for advancing our understanding of its mode of action, enhancing its clinical utility, and informing the development of next-generation microbiome-based therapeutics.

In the current study, we employed validated *in vitro* microbiota models, integrated with microbiome sequencing and targeted metabolomics, to investigate whether and how Sb mitigates the antibiotic-induced dysbiosis of the human gut microbiome. We implemented a two-step experimental framework. Initially, we screened the effects of amoxicillin/clavulanic acid (AMC, routinely used for many types of infection in human medicine) and vancomycin (Van, routinely given orally in the context of *Clostridioides difficile* infections), alongside Sb supplementation in the static MiPro model.^18^ While microbiome-stabilizing effects of Sb were observed under both antibiotic conditions, they were more consistently detectable and functionally robust in the context of AMC treatment. To explore these effects in greater detail, we employed the dynamic SHIME® model,^21^ which more closely recapitulates the spatial and temporal complexity of the human gut environment. To assess the host relevance of these microbiota-level changes, we performed *ex vivo* immune assays using human peripheral blood mononuclear cells (PBMCs) and intestinal mucosal explants exposed to microbiota-derived supernatants. Together, these approaches enabled us to characterize the functional impact of *S. boulardii* CNCM I-745 on the gut microbiome during antibiotic treatment and to evaluate its potential influence on host-relevant immune responses.

## Materials and methods

### Saccharomyces boulardii CNCM I-745 preparation

The probiotic Sb was provided as a lyophilized powder under GMP conditions by Laboratoires Biocodex (Gentilly, France) with a reported viability of >1.5 × 10⁷ colony-forming units (CFU) per milligram. The powder was stored at 4 °C. The viability of Sb was evaluated by plate counting on Sabouraud dextrose agar (#84088, Sigma-Aldrich) supplemented with chloramphenicol (50 mg/L, #C0378, Sigma-Aldrich).

### Ethics statement

The protocols of human stool, blood, and intestinal tissues collection were approved by the local ethics committee (Comité de Protection de Personnes Ile de France IV, IRB00003835 Suivitheque study; registration no. 2012/05NICB and Comité de Protection de Personnes Ile de France III, Biomhost study; EUDRACT number 2018-A02978-47-18.1). All human samples were collected with informed consent. Patients operated for colon adenocarcinoma (ADK) from the Biomhost study were recruited in the Department of Digestive Surgery at the Hospital Saint Antoine (Paris, France). Biopsy samples were collected in January 2025. Intestinal mucosa from tumor-free healthy margins was used.

### Human stool samples collection and preservation

Stool samples were collected from healthy volunteers aged 25 to 50. Exclusion criteria included diagnosed diseases, antibiotic use in the last three months, probiotic or prebiotic use in the last month, and pregnancy. Eight stool donors (four men (mean age: 39.8 y ± 6.4, mean BMI: 23.6 ± 1.8) and four women (mean age: 41.2 y ± 6.7, mean BMI: 22.1 ± 1.5)) were recruited in the MiPro study. Stool slurry aliquots (20% w/v) were prepared as described previously^22^ and stored at −80 °C until further use. Six stool donors (four men (mean age: 33.8 y ± 2.9, mean BMI: 22.2 ± 1.0) and two women (mean age: 45 y and mean BMI: 22.7) were recruited in the SHIME® study. Fresh stool slurries were prepared and inoculated into six parallel SHIME® systems as described previously.^23^

### Stool culturing in the static MiPro model and treatments

Stool stocks were thawed at 37 °C for approximately 10 min within an anaerobic workstation (5% H_2_, 5% CO_2_, 90% N_2_), then homogenized and inoculated into 1.2 mL MiPro culture medium at a final stool concentration of 2% (w/v). All steps were conducted under strict anaerobic conditions within the same chamber. The medium composition was described elsewhere.^18^ Static culturing was conducted in duplicate in 96-deep-well plates covered with Breathe-Easy® sealing membrane (#Z380059, Sigma-Aldrich) shaken at 650 rpm at 37 °C for 24 h in the anaerobic workstation. Each donor's sample was treated with antibiotics: AMC (Sandoz® 2 g amoxicillin/200 mg clavulanic acid) or Van (Mylan 1000 mg) at a final concentration of 0.1 mg/mL, and lyophilized Sb at a final concentration of 2 mg/mL (Sb+) or 4 mg/mL (Sb++), either separately or in combination. The applied concentrations of antibiotics and Sb were determined based on assumptions derived from clinical dosing normalized to colon content of 200 g^24^ and results from our pilot tests. Untreated samples were used as controls. Following culturing, samples were transferred to individual 2 mL Eppendorf tubes and centrifuged at 20,000 *g* for 10 min at 4 °C. The harvested pellets and supernatants were stored at −80 °C until further use.

### Stool culturing in the dynamic SHIME model and treatments®

SHIME® (ProDigest, Zwijnaarde, Belgium) was set up and operated as described previously.^25^ In brief, one SHIME® system consisted of one combined stomach and small intestine vessel (STSI), sequentially connected to proximal and distal colon compartments. The system was maintained at 37 °C using a warm water circulator (AC200, Thermo Fisher Scientific). Every 8 h, 140 mL of adult SHIME® growth medium (PD-NM001B, ProDigest) with pH 1.8−2.2 were pumped into the STSI. The medium contained 1.2 g/L arabinogalactan, 2 g/L pectin, 0.5 g/L xylan, 0.4 g/L glucose, 3 g/L yeast extract, 1 g/L special peptone, 3 g/L mucin, 0.5 g/L L-cysteine-HCl, and 4 g/L starch. It was sequentially digested with 60 mL of pancreatic juice, which consist of 12.5 g/L NaHCO3 (#S6014, Sigma-Aldrich), 6 g/L Oxgall (#11718223, Fisher Scientific), 0.9 g/L pancreatin (#P1625-100G, Sigma-Aldrich), before transfer to the colon compartments. The volumes of the proximal and distal colon vessels were kept at 500 and 800 mL, respectively. Their pH levels were controlled at 5.6−5.9 and 6.6−6.9 using built-in pH controllers and pumps adjusting the pH with 0.5 M NaOH or HCl.

**Figure 1.**
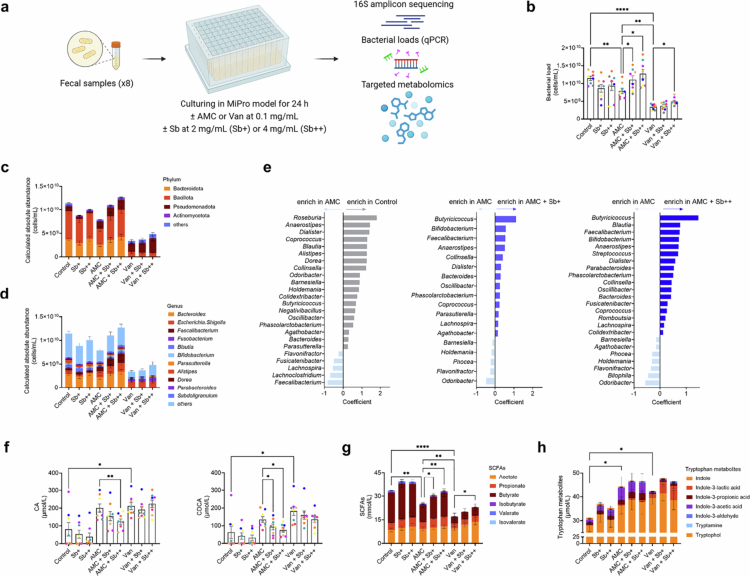
Sb supplementation ameliorates antibiotic-induced alterations in bacterial loads and metabolic outputs in a dose-dependent manner. (a) Schematic representation of *in vitro* culture of human fecal microbiota in the static MiPro model. Stool samples from eight healthy human donors were treated with AMC or Van (0.1 mg/mL) and supplemented with Sb (2 mg/mL or 4 mg/mL). The control group received no treatments. After 24 h of incubation, samples were collected for further analyses (Methods). (b) Bacterial load across different groups. (c),(d) Absolute abundance of bacteria at the phylum and genus level. Quantitative results were calculated by combining sequencing data with bacterial load measurements (Methods). Data were expressed as the mean ± standard error of the mean for each taxon. (e) Coefficient from MaAsLin analysis representing absolute abundances of genera with significant differences across different comparisons in AMC-treated samples. Only genera with a prevalence more than 49.9% and a q-value below 0.1 were shown. (f) Levels of primary bile acids. Cholic acid (CA) and chenodeoxycholic acid (CDCA) were present in the MiPro culture medium. (g) Levels of short-chain fatty acids. (h) Levels of tryptophan metabolites. Each color (b,f) represents one stool donor in MiPro. Levels of significance (b, f–h) were determined using paired one-way ANOVA followed by Bonferroni's post hoc test. *p < 0.05; **p < 0.01; ****p < 0.0001.

The experiment was conducted in three stages: a two-week stabilization period to establish the stable microbial community, followed by a one-week AMC treatment (50 mg three times a day, TID), and concluding with a two-week recovery period (Figure 3b). In the supplemented group (+Sb), lyophilized Sb (400 mg TID, 21 d) was added concurrently with AMC by direct addition to the autoclaved feed medium during both treatment and recovery phases. The control group (−Sb) received the same autoclaved feed medium containing AMC but without lyophilized Sb. The doses of AMC and Sb were based on clinical equivalents and pilot data, with the AMC dose adjusted to reflect 10% colonic bioavailability due to its high systemic absorption (~90%). Fermenter samples were collected daily at the same time since the start of the treatment, and centrifuged at 20,000 *g* for 10 min at 4 °C. The harvested pellets and supernatants were stored at −80 °C until further use Figure 1.

### Genomic DNA extraction

Pellets from MiPro and SHIME® experiments were subjected to DNA extraction following previously described methods.^26^ DNA concentration and quality were measured using a NanoDrop 2000 spectrophotometer (Thermo Fisher Scientific). DNA samples were stored at −80 °C until further use.

### Quantitative polymerase chain reaction measurement

Isolated DNA was subjected to quantitative polymerase chain reaction (qPCR) to quantify total bacterial sequences using TaqMan™ Universal PCR Master Mix (#4364340, Thermo Fisher Scientific) with the probe P_TM1389F 6FAM-CTTGTACACACCGCCCGTC and primers Bact1369-F CGGTGAATACGTTCCCGG and Prok1492-R TACGGCTACCTTGTTACGACTT. The subdominant bacterial groups were quantified using SYBR™ Green Universal Master Mix (#4364346, Thermo Fisher Scientific) with primers: Bact934-F GGARCATGTGGTTTAATTCGATGAT and Bact1060-R AGCTGACGACAACCATGCAG for Bacteroidota (formerly Bacteroidetes); Firm934-F GGAGYATGTGGTTTAATTCGAAGCA and Firm1060-R AGCTGACGACAACCATGCAC for Bacillota (formerly Firmicutes); Eco1457-F CATTGACGTTACCCGCAGAAGAAGC and Eco1652-R CTCTACGAGACTCAAGCTTGC for *Enterobacteriaceae*. Amplifications were conducted with an initial cycle at 95 °C for 10 min, followed by 40 cycles of 95 °C for 30 s and 60 °C for 1 min. For SYBR™ Green assays, a melting curve analysis step was added. All qPCR tests were performed 2 times independently. Data were calculated using standard curves generated from 10-fold serial dilutions of DNA from specific strains run in parallel with the experiment: *Escherichia coli* MG1655 for total bacteria and *Enterobacteriaceae*, *Bacteroides thetaiotaomicron* L55 for Bacteroidota, and *Blautia hansenii* DSM 20583 for Bacillota. The number of bacteria was determined using a microscope and a correlation between bacterial number and determined amount of DNA was established. Results were expressed as cells/mL.

### 16S rRNA amplicon sequencing and analysis

Isolated DNA from MiPro samples was subjected to PCR amplification using V3-V4 oligonucleotides (PCR1F_460: 5′-CTTTCCCTACACGACGCTCTTCCGATCTACGGRAGGCAGCAG-3′, PCR1R_460: 5′-GGAGTTCAGACGTGTGCTCTTCCGATCTTACCAGGGTATCTAATCCT-3′). Amplicon quality was verified by gel electrophoresis, and samples were sent to the @BRIDGe platform for the sequencing protocol on an Illumina MiSeq (Illumina, San Diego, CA, USA).

Raw paired-end reads were demultiplexed and quality filtered using QIIME2.^27^ Amplicon sequence variants (ASV) were created using DADA2.^28^ Taxonomic assignment was performed using SILVA database.^29^ Results were deep analyzed using *vegan* (v2.6-4, RStudio) and *phyloseq* (v1.34.0, RStudio) packages.^30^ Alpha diversity was presented by Shannon and Chao1 indexes. Beta diversity was visualized by principal coordinate analysis (PCoA) using the Bray–Curtis distance. Groupings were tested using PERMANOVA with 999 permutations and the *adonis2* function. Multivariable association between microbial community abundance and treatment was examined using MaAsLin2 with donor as a covariate.^31^ Plotting was performed using *ggplot2* (v3.5.0, RStudio).

### Shotgun metagenomic sequencing and analysis

Isolated DNA from SHIME® samples was subjected to shotgun metagenomic sequencing performed at Prebiomics (Trento, Italy). In brief, DNA was quantified using Quant-iT™ 1X dsDNA Assay Kits (#Q33267, Life Technologies) and diluted in water. The sequencing libraries were prepared with the Illumina DNA Prep kit (#20060059, Illumina) and Illumina UD Indexes (#20091648, Illumina) according to the manufacturer's protocol, and sequenced on the Novaseq X Plus platform at an average depth of 7.5 Gb raw data per sample.

Raw sequencing reads were subjected to quality assessment using FastQC v0.12.1 and subsequently filtered to remove low-quality sequences and adapters using Cutadapt v4.9. To eliminate human-derived contaminants, reads were aligned against the reference human genome (GRch38) using Bowtie2 v2.5.4. Processed reads were then analyzed through two parallel workflows: (1) filtration against the Sb reference genome (GCA_001298375.2), and (2) direct analysis of all non-human reads. Taxonomic profiling was performed using MetaPhlAn v4.1.1.^32^ Functional profiling was conducted using HUMAnN v3.9^33^ which estimates MetaCyc pathways and UniRef90 gene families' abundances from metagenomic reads. Results were analyzed using the *vegan* package (v2.6-4, RStudio). Alpha diversity was presented by Shannon and Chao1 indexes. Beta diversity was visualized using PCoA with centered log-ratio (CLR) transformation and Aitchison distance. Half of the minimum proportional abundance was used for the imputation of zeros in CLR. Differences in the overall microbiota composition were statistically evaluated using PERMANOVA with 999 permutations. Multivariable association between microbial community abundance and treatment was examined using MaAsLin2/3 with donor as a covariate.^31^

### Antibiotic resistance gene profiling

To determine the composition and abundance of antibiotic resistance genes (ARGs) in each sample, high-quality cleaned reads, excluding human and Sb-aligned sequences, were analyzed using ResFinder v2.4.0^34^ for culturable and pathogenic bacteria and ResFinderFG v2.0^35^ for non-culturable and non-pathogenic bacteria. Subsampled datasets were processed with ARGs-OAP v3.2.4^36^ to obtain the annotation of ARG profiles. The relative abundance of ARGs was calculated from metagenomic datasets following previously established methodologies.^36^ Identified ARGs were functionally annotated and classified according to their targeted antibiotic classes (e.g., tetracycline, *β*-lactam) or specific antibiotics (e.g., AMC, Van). ARGs conferring resistance to AMC, identified through ResFinder and ResFinderFG, were integrated for further analysis. All tools were applied with default settings to ensure consistency, and ARG classifications were cross-referenced with standardized resistance gene nomenclature to maintain accuracy in functional categorization.

### Quantitative microbiota profiling

A quantitative microbiota profiling matrix was constructed as previously described.^37^ Sequencing-based taxon counts (from 16S rRNA gene amplicon or shotgun metagenomic data) were first normalized to relative abundances by dividing each taxon count by the total read count per sample. To convert these relative profiles into quantitative microbiota profile (QMP), the relative abundance of each taxon was multiplied by the total bacterial load of the corresponding sample, which was quantified using universal targeted qPCR. This approach enables estimation of absolute taxon abundances by integrating relative sequencing data with absolute bacterial biomass measurements. Importantly, it corrects for potential biomass contributions from the supplemented probiotic (Sb), allowing for more accurate assessment of bacterial community dynamics under treatment conditions.

### Targeted metabolomics

Culture supernatants of MiPro and SHIME® samples underwent targeted metabolomics in house or at the core facility PMAC of the US61 ASB (Université de Tours, France) to quantify short-chain fatty acids (SCFAs) and tryptophan metabolites following previously described methods.^38-40^ Bile acids (BAs) were measured on a Waters® ultra-high-performance liquid chromatography system coupled with a Waters® Xevo TQ-XS Triple Quadrupole Mass Spectrometer. Chromatographic separation was performed using mobile phase A: 1 mM ammonium formate and 0.1% formic acid in 10% acetonitrile and mobile phase B: 1:1 acetonitrile: isopropanol on a Waters® ACQUITY BEH C8 (2.1 × 100 mm, 1.7 µm) column maintained at 60 °C. Fermenter supernatant (50 µL) was mixed thoroughly with 450 µL of methanol, centrifuged at 4500 *g* for 30 min at 4 °C, and then 50 µL of the resulting supernatant was mixed thoroughly with 250 µL of 50% methanol, followed by transfer of 50 µL to a 96-well plate and addition of 50 µL internal standard solution for analysis. The injection volume was 2 µL. The gradient elution began at 90% mobile phase A for 0.1 min, decreased to 65% by 9.25 min, to 15% by 11.5 min, and to 0% from 11.8 to 12.4 min, then increased to 45% by 12.45 min and returned to 90% from 12.5 to 14 min. The flow rate was adjusted throughout the run, starting at 0.6 mL/min for 9.25 min, increasing to 0.65 mL/min over 2.25 min, to 0.8 mL/min over 0.3 min, to 0.95 mL/min over 0.2 min, to 1 mL/min over 0.1 min and held for 0.3 min, then decreasing to 0.85 mL/min over 0.05 min and held for 0.05 min, to 0.8 mL/min over 0.1 min, to 0.7 mL/min over 0.1 min, finally to 0.6 mL/min over 0.1 min and held until the end of the run. Bile acid identification and processing were done with TargetLynx® (Waters) software. Quantification was performed against a standard curve for each bile acid, normalized to internal standards. BAs in the SHIME® samples were not quantified as they are present in pancreatic juice, which was continuously supplied to support digestion.

### Human PBMCs isolation and stimulation

Whole blood from four healthy males donors (mean age: 31.0 y ± 3.2, mean BMI: 22.4 ± 1.2) was 1: 1 mixed with dilution buffer: PBS containing 1% fetal bovine serum (FBS) (#17479633, Gibco) and 5 mM EDTA (#15575020, Thermo Fisher Scientific), then transferred to SepMate™-50 tubes (#85450, STEMCELL) pre-filled with 14 mL of Histopaque−1077 (#10771, Sigma-Aldrich), and centrifuged at 1200 g for 10 min. The upper phase, containing PBMCs, was collected and washed with dilution buffer. The cell pellets were treated with 1X Red Blood Cell lysis buffer (#420301, Biolegend) and centrifuged at 300 g for 5 min. Isolated PBMCs were counted and re-suspended at 5.6 × 10^5^ cells/mL in PBMC culture medium: RPMI 1640 GlutaMAX™ medium (#72400047, Gibco) supplemented with 10% FBS (#17479633, Gibco), 1% Non-Essential Amino Acid (NEAA) (#11140050, Thermo Fisher Scientific), 100 U/mL Pen-Strep (#11548876, Gibco), and 0.1 mM pyruvate (#12539059, Gibco).

PBMCs were seeded into 96 well U-bottom Plates (#7007, Corning) at a volume of 180 µL per well and stimulated in duplicate with 20 µL of PBS, lipopolysaccharide (LPS; 1000 ng/mL, (#tlrl-eklps, Invivogen)), and MiPro/SHIME® supernatants for 24 h in a humidified incubator set to 37 °C with 5% CO_2_. Following incubation, cells were removed by centrifugation at 500 *g* for 5 min. The cell supernatants were immediately analyzed for cytotoxicity using Cytotoxicity Detection Kit^PLUS^ (LDH) (#11644793001, Roche) according to the manufacturer's instructions and stored at −80 °C until further use.

### Human intestinal mucosa collection and stimulation

Intestinal mucosal tissues were obtained from tumor-free margins of surgically resected colon specimens from two patients diagnosed with ADK (a 70-y-old man and a 67-y-old woman) and washed three times for 5 min each in an antibiotic and antifungal cocktail: 100 U/mL Pen-Strep (#11548876, Gibco), 100 μg/mL gentamicin (#11500506, Gibco), and 0.1 μg/mL amphotericin B (#15290018, Gibco). Fragments of 2 mm^2^ were cut and placed into 48-well plates with 450 µL of PBMC culture medium per well, and stimulated with 50 µL of PBS and SHIME® supernatants in duplicate or triplicate depending on the number of fragments, then incubated for 16 h in a humidified incubator set to 37 °C with 5% CO_2_. Following incubation, the culture supernatants were collected by centrifugation at 500 *g* for 5 min, and immediately analyzed for cytotoxicity using Cytotoxicity Detection Kit^PLUS^ (LDH) (#11644793001, Roche) according to the manufacturer's instructions and stored at −80 °C until further use. The mucosal samples were subjected to total protein quantification using BC Assay Protein Quantitation Kit (#UP40840A, Interchim) according to the manufacturer's instructions. In brief, intestinal mucosa was treated with 400 µL of RIPA lysis and protein extraction buffer (#89900, Thermo Fisher Scientific), supplemented with 1X protease inhibitor cocktail (#11697498001, Roche), and incubated for 45 min on ice with occasional vortexing to enhance protein solubilization. Next, samples were centrifuged at 20,000 *g* for 10 min, and the supernatant was transferred for protein quantification.

### Cytokine quantification

ELISAs were conducted to quantify TNF-*α* according to the manufacturer's instructions (#88-7346, Invitrogen). A complex set of cytokines was quantified using LEGENDplex™ Human Inflammation Panel 1 (#740809, Biolegend) according to the manufacturer's protocol, analyzed on a BD Accuri™ C6 flow cytometer and quantified using LEGENDplex™ analysis software (Biolegend). The results were normalized to LDH data, with mucosal samples further normalized to protein content.

### Data and statistical analysis

Data are expressed as the mean ± standard error of the mean. Stool donors in each study represent biological replicates. PBMCs isolated from four healthy donors and mucosal samples isolated from two ADK patients were used as technical replicates. Results were consistent across donors and averaged for analysis. Individual donor data were presented in the supplementary figures. GraphPad Prism v 10.4.0 was used for the analyses and preparation of graphs. To compare three or more related groups, paired one-way ANOVA was used followed by Bonferroni's post hoc test to correct for multiple comparisons. For analyses involving two factors (e.g., treatment and time), paired two-way ANOVA was used with Bonferroni's correction. Where applicable, paired two-tailed Wilcoxon tests were used for nonparametric comparisons between two related groups. Details of the statistical tests applied to each figure are provided in the figure legends. Only biologically relevant and statistically meaningful comparisons are highlighted in the figures. Differences were considered statistically significant at *p* < 0.05.

## Results

### Sb supplementation does not impact antibiotic-induced alterations in taxonomic composition

To evaluate the effect of Sb supplementation on antibiotic-induced microbiome alterations, we conducted stool culture experiments in the MiPro system, a 96-deep well plate-based culturing model that can preserve the compositional and functional characteristics of individual human gut microbiome.^18^ We first evaluated Sb's growth potential by culturing Sb in MiPro medium under anaerobic conditions. Sb maintained viability but exhibited no net growth at 24 h, indicating that MiPro medium did not support active proliferation of Sb under anaerobic conditions (Figure S1a). We tested two commonly prescribed antibiotics, AMC and Van, each at a final concentration of 0.1 mg/mL, alongside Sb supplementation at a final concentration of 2 or 4 mg/mL, on fecal microbiota from eight healthy human donors (Figure 1a). After 24 h of treatment, both antibiotics significantly reduced alpha diversity as measured by the Shannon index, but had no significant effect on species richness (Chao1 index; Figure S2a). This reduction in diversity was accompanied by significant shifts in community composition compared to the control group (PERMANOVA, *p* = 0.007 for AMC, *p* = 0.001 for Van; Figure S2b), as revealed by PCoA of beta diversity. Additionally, beta diversity analysis pointed out significant differences between the two antibiotic-treated groups (PERMANOVA, *p* = 0.001; Figure S2b), indicating distinct effects of AMC and Van on the gut microbiome. Stool-derived *in vitro* microbial communities were dominated by Bacillota and Bacteroidota phyla (Figure S2c). AMC-treated samples exhibited disruptions in microbiota composition, with an increase in the relative abundance of the phylum Pseudomonadota (formerly Proteobacteria) and a decrease in the relative abundance of the phylum Bacillota. These shifts were more pronounced in Van-treated samples (Figure S2c). Consistent with previous studies reporting that microbial interactions in complex communities modulate antibiotic efficacy,^41^^,^^42^ we found that Van,despite its specificity for Gram-positive bacteria,^1^ led to a distinct loss of several Gram-negative commensals, including *Bacteroides* and *Parabacteroides* (Figure S2d-e). Sb supplementation, regardless of the dose and antibiotic treatment, had no significant effect on antibiotic-induced alterations in taxonomic composition (Figure S2).

**Figure 2.**
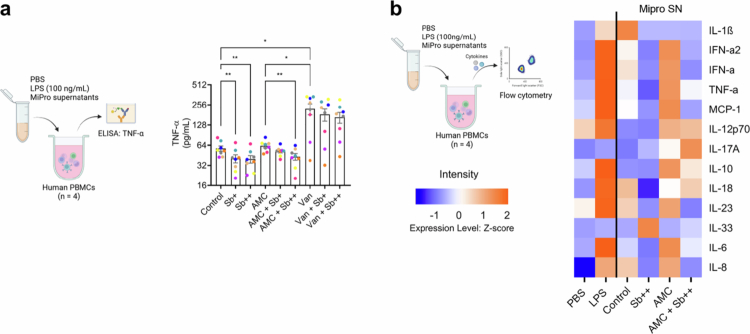
Effects of Sb supplementation on the gut microbiome are associated with a reduced pro-inflammatory potential. (a) Levels of TNF-α after stimulation with MiPro supernatants in human PBMCs. Each color represents one stool donor in MiPro. (b) Heatmap visualization of inflammatory cytokine levels, scaled by Z-scores, after stimulation with representative MiPro culture supernatants in PBMCs isolated from four healthy donors. PBS and LPS columns display average cytokines levels from PBMCs stimulated with PBS and LPS (100 ng/ml), while other columns display average data from these PBMCs stimulated with eight stool donors in MiPro. The color in the heatmap represents high (orange) or low (blue) cytokine levels. Results were consistent across PBMC donors and averaged for analysis. Individual donor data are presented in Figure S6. Levels of significance (a) were determined using paired one-way ANOVA followed by Bonferroni's post hoc test. *p < 0.05; **p < 0.01.

### Sb supplementation ameliorates antibiotic-induced alterations in bacterial loads and metabolic outputs in a dose-dependent manner

Since antibiotics affect the gut microbiome both qualitatively and quantitatively^6^, we next examined the effects of Sb supplementation on bacterial loads determined by qPCR. This approach eliminates potential interference from the biomass introduced by Sb supplementation. As expected, both antibiotics significantly reduced bacterial load, with Van having a more pronounced effect (Figure 1b). In AMC-treated samples, Sb supplementation counteracted the antibiotic's effect in a dose-dependent manner, while a milder restorative effect was observed in Van-treated samples (Figure 1b). Specifically, Sb supplementation reversed the AMC-induced reduction in the population of Bacteroidota and Bacillota, which were dramatically reduced in Van-treated samples (Figure S3a,b). A non-significant increase in *Enterobacteriaceae* was observed under Van treatment with Sb supplementation (Figure S3c).

Next, we integrated sequencing data with bacterial load measurements to generate QMP. This approach provides precise insights into the extent of changes in bacterial abundance, thereby reducing the risk of erroneous interpretations of microbiome associations.^43^ Unlike relative microbiota profiles, Sb supplementation markedly reshaped the QMP, in which it partially reversed the AMC-induced perturbations at the phylum level and significantly increased the absolute abundances of several genera, including *Bacteroides*, *Faecalibacterium*, and *Bifidobacterium*, compared to non-supplemented samples (Figure 1c-e). Van inactivated a large number of bacteria, resulting in limited effects with Sb supplementation on the QMP (Figure 1c, d).

Given the crucial role of metabolites in the effect of the gut microbiome on the host, we performed targeted metabolomics to quantify three of the main families of microbiome-derived metabolites: BAs, SCFAs, and tryptophan metabolites. We first measured microbial metabolite production by Sb incubated in MiPro medium under anaerobic conditions at 24 h (Figure S1b). Metabolite levels were similar to those of uninoculated media and substantially lower than in fecal microbiota–inoculated cultures (0.3 vs 30 mmol/L; Figure S1b), indicating minimal metabolic activity by Sb under these conditions. These findings suggest that, under the tested *in vitro* conditions, Sb contributes minimally to the overall metabolic output. Compared to controls, antibiotic-treated samples had lower levels of SCFAs, and higher levels of primary BAs (cholic acid, CA; chenodeoxycholic acid, CDCA) and tryptophan metabolites, particularly indole (Ind) (Figure 1f-h). This latter finding may result from changes in bacterial loads and taxonomic features, such as an increased abundance of *Enterobacteriaceae* (Figure S3c), known to produce Ind.^44^ Consistent with previous reports,^45^ Sb supplementation promoted microbial conversion of CA and CDCA, along with increased production of SCFAs (propionate and butyrate) and indole-3-propionic acid (IPA) in AMC-treated samples in a dose-dependent manner (Figure 1f-h and Figure S4). At a higher concentration of AMC (0.5 vs 0.1 mg/mL), the protective effects of Sb supplementation were no longer observed, likely due to the excessive bacterial loss (Figure S5). Under Van treatment, Sb supplementation led to elevated levels of acetate and, notably, indole-3-lactic acid (ILA; Figure S4). Together, these results indicate that Sb supplementation protects against antibiotic-induced dysbiosis by recovering bacterial load leading to metabolic functions recovery.

### Effects of Sb supplementation on the gut microbiome are associated with a reduced pro-inflammatory potential

To evaluate the potential effect of the observed microbiome alterations on host immune responses, we stimulated human PBMCs from four healthy donors with MiPro supernatants and quantified cytokine production. To ensure assay responsiveness and account for donor variability, LPS (100 ng/mL) was included as a positive control and elicited robust cytokine responses across all donors, confirming assay integrity (Figure 2 and Figure S6a). In addition, PBMCs were stimulated with culture supernatants from Sb monocultures grown anaerobically for 24 h in MiPro medium. These supernatants did not consistently alter cytokine secretion profiles relative to blank media controls across donors (Figure S6a). Compared to untreated controls, stimulation with antibiotic-treated samples led to increased secretion of tumor necrosis factor-alpha (TNF-*α*), a key pro-inflammatory cytokine (Figure 2a). Sb supplementation significantly reduced TNF-*α* production in both control and AMC-treated conditions. To expand the analysis, we performed multiplex profiling of inflammation-related cytokines using a customized human inflammation panel. AMC-treated samples induced elevated levels of several pro-inflammatory cytokines, including TNF-*α*, interferon alpha-2 (IFN-α2), IFN-*γ*, interleukin-23 (IL-23), IL-6, IL-8, and Monocyte Chemoattractant Protein-1 (MCP-1), relative to untreated controls (Figure 2b). Sb supplementation partially attenuated these elevations, suggesting a potential modulatory effect on the immune-stimulatory capacity of the microbiome. Donor-specific cytokine profiles demonstrated consistent trends in the direction and magnitude of Sb's modulatory effects, despite expected inter-individual variation (Figure S6b,c). Collectively, these results demonstrate that the mitigation of antibiotic-induced microbiome alterations by Sb supplementation may have effects on the host with a reduced pro-inflammatory potential.

**Figure 3.**
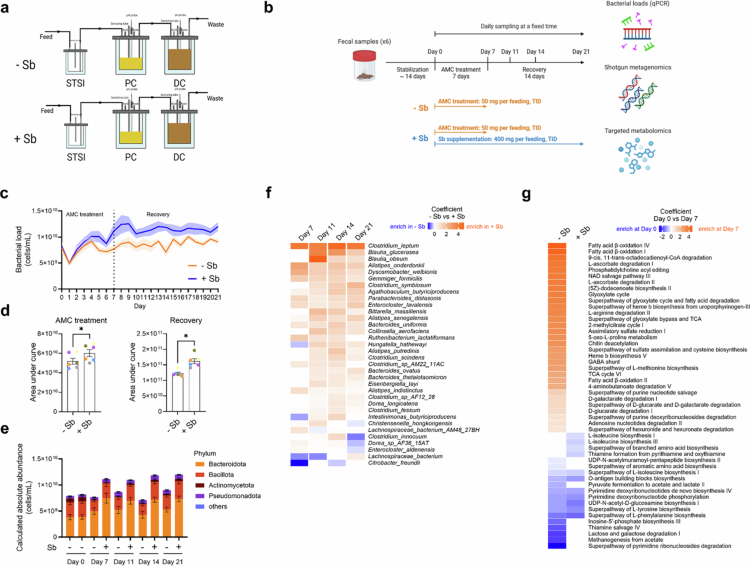
Sb supplementation induces protective effects against AMC-induced gut microbiome alterations in the dynamic SHIME^®^ model. (a) Schematic representation of the dynamic SHIME^®^ model. Stool samples were cultured in proximal (PC) and distal colon (DC) compartments with continuous feeding (Methods). (b) Schematic representation of long-term intervention of AMC and Sb. Stool samples from 6 independent healthy human donors were sequentially inoculated and stabilized for two weeks. Sb was supplemented in the feed (400 mg TID for 21 d) during and after AMC treatment (50 mg TID for 7 d) (+Sb), while a control group received AMC alone (−Sb). After stabilization, samples were daily collected at a fixed time for further analyses (Methods). (c),(d) Bacterial load in DC compartments throughout the experiment and the area under the curve (d). Each color (d) represents one stool donor in SHIME^®^. Levels of significance (d) were determined using paired two-tailed Wilcoxon test. *p < 0.05. (e) Absolute abundance of bacteria at the phylum level. Data were expressed as the mean ± standard error of the mean for each taxon. (f) Heatmap visualization of coefficient from MaAsLin analysis representing absolute abundances of species with significant differences between with Sb supplementation (+Sb) and without Sb supplementation (−Sb) at different time points. Only species with a prevalence more than 49.9% and a q-value below 0.1 were shown. (g) Heatmap visualization of coefficient from MaAsLin analysis representing MetaCyc pathways with significant differences between Day 7 with or without Sb supplementation (+Sb or −Sb) and Day 0. Only MetaCyc pathways with a prevalence more than 49.9% and a q-value below 0.25 were shown.

### Sb supplementation induces protective effects against AMC-induced gut microbiome alterations in the dynamic SHIME model®

To extend our findings from the MiPro model to a more physiologically relevant system, we employed SHIME®, a multi-compartment dynamic simulator of the gastrointestinal tract that enables long-term, automated, and stable cultivation of the human gut microbiome.^21^ We selected AMC for further investigation as it induced more pronounced and consistent microbiome disruption than Van in the MiPro model. Moreover, AMC is a widely prescribed broad-spectrum antibiotic frequently associated with gut dysbiosis and antibiotic-associated diarrhea, reinforcing its relevance for in-depth analysis. In SHIME® medium, Sb remained viable but did not exhibit net growth (Figure S1a). Fresh stool samples from six independent healthy human donors were sequentially inoculated into SHIME®, which was designed to examine microbial dynamics along the colon (Figure 3a), with a particular focus on the distal colon (DC), as the stool microbiota primarily originates from this region. After a two-week stabilization, Sb was supplemented in the feed (400 mg TID for 21 d) during and after AMC treatment (50 mg TID for 7 d), while a control group received AMC alone (Figure 3b). Shotgun metagenomics was performed on longitudinal samples to investigate dynamic changes in the gut microbiome. Prior to analysis, sequences annotated to Sb were removed because they induced bias in functional profiling (Figure S7). AMC treatment significantly reduced microbial alpha diversity, as reflected by decreases in both Shannon and Chao1 indices (Figure S8a). Sb supplementation partially reversed this reduction, with a more pronounced effect on species richness (Chao1 index), and supported a slightly improved recovery in Chao1 compared to the non-supplemented group (Figure S8a). Beta diversity did not differ significantly between groups, nor did relative microbiota profiles, but strong subject-specific clustering was observed (Figure S8b-d). Taxonomic analysis of relative abundance at the species level revealed distinct, personalized gut microbiota profiles (Figure S8e), suggesting that the SHIME® model effectively captured donor specificity. Donor-dependent variations were observed in microbiota responses to AMC and Sb, with inter-individual differences outweighing treatment-induced changes in the relative abundance (Figure S8e).

Additionally, bacterial load was tracked longitudinally by qPCR and the area under the curve was calculated to quantify the changes in microbial dynamics. Sb supplementation promoted microbial recovery following AMC perturbation with a notable increase in bacterial load (Figure 3c,d), particularly within the Bacteroidota and Bacillota phyla (Figure S9a,b), an observation consistent with findings from the MiPro model. AMC treatment initially caused a decline in bacterial load, but an overgrowth of bacteria was subsequently observed (Figure 3c), including *Enterobacteriaceae*, which was partially inhibited by Sb supplementation (Figure S9c). Next, we assessed the dynamic changes in the QMP. Consistent with qPCR measurements, Sb supplementation increased the absolute abundances of Bacteroidota following AMC perturbation (Figure 3e). Notably, Sb supplementation selectively favored the growth of certain species, known to promote intestinal and metabolic health, including *Clostridium leptum,*^46^ *Dysosmobacter welbionis,*^47^ *Parabacteroides distasonis,*^48^ *Bacteroides* uniformis,^49^ and several *Alistipes* spp.^50^ (Figure 3f).

To determine changes in microbial metabolic functionality, we conducted metagenomic functional analysis using HUMAnN and the MetaCyc database. MetaCyc pathway distributions exhibited greater homogeneity across subjects compared to taxonomic composition (Figure S10a), a pattern consistent with functional redundancy^51^. Differential abundance analysis revealed 46 MetaCyc pathways with significant alterations in relative abundance between Day 0 (baseline) and Day 7 (end of AMC treatment), whereas Sb supplementation reduced the number of AMC-perturbed pathways to 11 (Figure 3g). Many of these differentially abundant pathways were associated with essential bacterial metabolic processes, including energy production and carbohydrate degradation. Direct cross-group comparisons between supplemented and non-supplemented groups at Day 7 further confirmed Sb-mediated functional remodeling (Figure S10b), highlighting Sb's role in mitigating AMC-induced functional dysbiosis. Taken together, these results confirm the protective effects of Sb supplementation against AMC-induced dysbiosis in dynamic settings.

### Sb supplementation stimulates propionate and IPA production and mitigates AMC-induced functional dysbiosis

**Figure 4.**
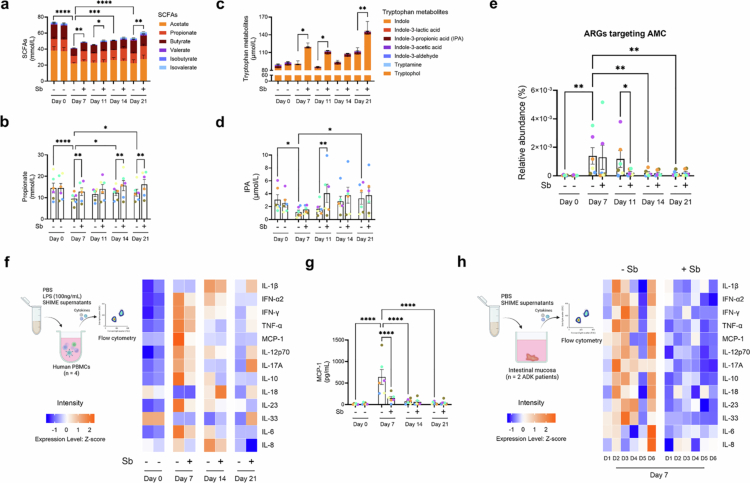
Sb supplementation stimulates propionate and IPA production in the dynamic SHIME® model and attenuates pro-inflammatory effects associated with AMC-perturbed gut microbiome. (a) Levels of short-chain fatty acids. (b) Levels of propionate. (c) Levels of tryptophan metabolites. (d) Levels of indole-3-propionic acid (IPA). (e) Levels of antibiotic resistance genes (ARGs) targeting AMC. (f) Heatmap visualization of inflammatory cytokine levels, scaled by Z-scores, after stimulation with representative SHIME® supernatants in human PBMCs. Each column displays average data from 6 stool donors in SHIME®. (g) Levels of Monocyte Chemoattractant Protein-1 (MCP-1) obtained as described in (f). (h) Heatmap visualization of inflammatory cytokine level, scaled by Z-scores, after stimulation with SHIME® culture supernatant at Day 7 in human intestinal mucosa. Each column displays data from one stool donor in SHIME®. The color in the heatmap (f,h) represents high (orange) or low (blue) cytokine levels. PBMCs isolated from four healthy donors and mucosal samples isolated from 2 ADK patients served as technical replicates. Results were consistent across donors and averaged for analysis. Individual donor data are presented in Figure S12 and Figure S13. Each color (b,d,e,g) represents one stool donor in SHIME®. Levels of significance (b,d,e,g) were determined using paired two-way ANOVA with Bonferroni's post hoc test. *p < 0.05; **p < 0.01; ***p < 0.001; ****p < 0.0001.

To examine the impact of Sb supplementation on microbiota metabolic output, we measured the production of SCFAs and tryptophan metabolites. Of note, levels of SCFA and Trp metabolites in 24h-culture of Sb in SHIME® medium were similar to that of uninoculated medium and substantially lower than in fecal microbiota–inoculated cultures (Figure S1c), indicating minimal metabolic activity by Sb under these conditions. AMC treatment significantly reduced SCFAs levels, while Sb supplementation partially restored their levels during the treatment and recovery phases (Figure 4a). In particular, Sb supplementation promoted the microbial production of propionate (Figure 4b), a metabolite predominantly synthesized by Bacteroidota with substantial effects on mucosal and systemic immune responses^52^. Tryptophan metabolites were dominated by Ind (Figure 4c), whose levels reflected changes in overall bacterial abundance, as it is broadly produced via tryptophanase activity among gut taxa.^44^ Of note, Sb supplementation enhanced the microbial synthesis of IPA (Figure 4d), a microbiota-derived metabolite with immunomodulatory properties.^53^ These results are consistent with those observed in the MiPro model and further support the role of Sb in protecting community function by maintaining bacterial load under antibiotic stress.

Given that antibiotic exposure not only disrupts microbiota composition and function but also promotes the expansion of antibiotic resistance genes (ARGs; the resistome),^54^ we next assessed resistome dynamics across treatment groups. This represents a key advantage of dynamic microbiota models such as SHIME®, which enable longer-term ecological monitoring. As expected, AMC treatment induced a significant increase in the relative abundance of AMC-resistant ARGs (Figure 4e). Sb supplementation partially attenuated this enrichment in most donors by Day 7, with a statistically significant reduction observed by Day 11 (Figure 4e). Correlation analysis showed a positive association between relative abundances of AMC-resistant ARGs and *Enterobacteriaceae* (Figure S11a). Consistent with this, Sb also partially suppressed AMC-induced expansion of *Enterobacteriaceae* in most donors, with significant differences likewise emerging at Day 11 (Figure S11b).

### Sb supplementation reduces pro-inflammatory cytokines production induced by the gut microbiota in human PBMCs and intestinal mucosa

To assess the immunomodulatory effects associated with Sb-induced metabolic changes, human PBMCs from four donors were stimulated with SHIME® supernatants, and cytokine production was measured using multiplex analysis. LPS (100 ng/mL) was included as a positive control and induced robust cytokine responses across all donors (Figure S12a), confirming assay responsiveness. In addition, PBMCs were stimulated with culture supernatants from Sb monocultures grown anaerobically for 24 h in SHIME® medium. These supernatants did not consistently alter cytokine secretion profiles relative to blank media controls across donors (Figure S12). AMC-perturbed gut microbiota at Day 7 demonstrated an increased pro-inflammatory potential, reflected by elevated levels of multiple pro-inflammatory cytokines relative to baseline (Day 0) (Figure 4f). Sb supplementation attenuated this response, with significantly reduced levels of MCP-1 and other pro-inflammatory cytokines compared to non-supplemented AMC-treated samples (Figure 4f,g). To further validate these findings in a physiologically relevant system, human colon mucosal explants obtained from tumor-free margins of ADK patients (*n* = 2 donors) were stimulated with Day 7 SHIME® supernatants. No modification of cytokine secretion profiles was observed when mucosal explants were stimulated with culture supernatants from Sb monocultures grown anaerobically for 24 h in SHIME® medium compared to SHIME® medium alone (Figure S13a). However, similar to PBMCs, pro-inflammatory cytokine levels were lower following stimulation with Sb-supplemented samples compared to non-supplemented controls (Figure 4h). Donor-specific cytokine profiles from both PBMC and mucosal assays are presented in the supplementary figures (Figures S12b and S13), demonstrating consistent immunomodulatory trends despite inter-individual variation. In conclusion, these results indicate that Sb supplementation mitigates AMC-induced gut microbiome alterations in the SHIME® model and the associated pro-inflammatory host responses.

## Discussion

Extensive clinical data have established *Saccharomyces boulardii* CNCM I-745 as an effective adjunct during antibiotic treatment, with documented benefits mediated through direct interactions with the gut epithelium.^55^ These include immune modulation via Toll-like receptor signaling and enhancement of epithelial barrier integrity through upregulation of tight junction proteins.^55^ Sb also exhibits intrinsic metabolic activity, including the production of bioactive metabolites such as SCFA,^15^ which may further contribute to intestinal homeostasis. Furthermore,it has been demonstrated that Sb does not alter the pharmacokinetics of AMC.^56^ However, the extent to which Sb directly modulates the structure and function of the antibiotic-perturbed gut microbiome—independent of host-mediate feedback^57^—remains incompletely understood.

To fill this knowledge gap, we employed validated *in vitro* microbiota models and systematically evaluated the impact of Sb on microbiome composition and function under antibiotic stress. In these models, the magnitude of microbiome perturbations can be tuned both qualitatively and quantitatively.^23^^,^^58^ This is particularly relevant in light of growing evidence implicating microbial biomass as a key determinant of microbiome function in health and disease,^37^^,^^43^^,^^59^^,^^60^ Here, we demonstrated that Sb-mediated microbiome protection against AMC occurred primarily through the stabilization of bacterial biomass, with inconsistent effects on taxonomic structure. This contrasts with other *in vitro* studies, where probiotics directly reshape microbial composition.^61-63^ One likely explanation is the substantial inter-individual variability observed across donors, which may mask consistent group-level compositional shifts. While our sample sizes—eight donors for MiPro and sixfor SHIME®—are relatively large compared to many *in vitro* microbiome studies (often using three donors), they remain insufficient to capture the full spectrum of human microbiome diversity. Despite the absence of significant taxonomic restructuring, Sb supplementation led to marked and reproducible alterations in metabolite profiles under AMC treatment. This apparent discrepancy may reflect biomass-driven metabolic activity, where Sb preserved or increased the absolute abundance of key functional taxa. In contrast, under Van treatment, Sb had minimal impact on bacterial biomass or taxonomic composition. These findings reinforce that probiotic efficacy is not uniform across antibiotic classes and should be evaluated in an antibiotic-specific context.

While inter-individual taxonomic responses to Sb were variable, functional pathway profiles remained comparatively stable across donors. This pattern is consistent with the concept of functional redundancy in the gut microbiome,^64^ wherein distinct microbial taxa can perform overlapping metabolic functions, thereby buffering community-level functionality against compositional perturbations. In this context, Sb supplementation was associated with reduced disruption of metabolic pathways under AMC treatment, particularly those related to carbohydrate degradation, stress adaptation, and energy metabolism. Notably, these functional effects occurred despite minimal and inconsistent taxonomic shifts, suggesting that the preservation of microbial function does not necessarily require major changes in community composition. The observed functional resilience may thus reflect the ability of Sb to support microbial biomass and sustain core metabolic processes under antibiotic pressure. Together, these results underscore the importance of incorporating functional assessments alongside taxonomic analyses when evaluating microbiome stability, and highlight functional redundancy as a potential contributor to the consistent mitigation of dysbiosis observed across donors.

SCFA induction is a well-documented effect of many probiotics,^65^ and in our study, Sb supplementation promoted SCFA production, particularly propionate under AMC treatment. This effect likely reflects enhanced activity of SCFA-producing taxa such as *Bacteroides* spp.^52^, whose absolute abundance increased in Sb-treated samples,potentially supported by the fermentable properties of Sb's cell wall.^66^ In addition to SCFAs, we observed enrichment of tryptophan-derived metabolites, IPA under AMC treatment and ILA under Van treatment, both of which have recognized immunomodulatory properties.^67-70^ Unlike bacterial probiotics such as *Lactobacillus reuteri,*^71^ which can convert tryptophan via encoded indoleamine 2,3-dioxygenase, Sb lacks the genetic capacity for direct tryptophan metabolism. These shifts are therefore likely mediated through ecological facilitation of endogenous tryptophan-degrading microbes, such as *Clostridium* spp.^72^ This interpretation is supported by previous *in vitro* and *in silico* studies showing that Sb can promote mutualistic microbial interactions and enhance the production of functionally beneficial metabolites.^73^

Our resistome analysis suggests that Sb supplementation may modestly attenuate AMC-induced enrichment of resistance genes, potentially via partial suppression of *Enterobacteriaceae*, a known reservoir of *β*-lactamase–encoding ARGs.^74^ This observation is consistent with prior human studies reporting Sb-associated reductions in *Enterobacteriaceae* during AMC treatment.^13^ However, these effects were limited in magnitude and only became statistically significant at later time points. Notably, no comparable reduction in *Enterobacteriaceae* was observed under Van exposure, where Sb supplementation was associated with increased abundance. These findings suggest that Sb's modulatory effects on ARGs may not be uniform across antibiotic classes. Further studies and broader comparative analyses are warranted to determine the contexts in which such interventions may effectively support resistome management.

A key strength of this study lies in the integration of microbiome modulation with host immune responses, a link often overlooked in probiotic research.^75^^,^^76^ Using *ex vivo* human PBMCs and intestinal mucosal explants, we connected Sb-associated microbiome remodeling under AMC treatment to reduced pro-inflammatory cytokine secretion, potentially mediated by increased levels of immunomodulatory metabolites such as propionate and IPA. Propionate enhances immune tolerance through the promotion of regulatory T cells,^77^while IPA suppresses NF-κB-driven inflammatory signaling.^78^ Importantly, Sb monocultures incubated anaerobically in MiPro and SHIME® media showed minimal metabolic activity, and their supernatants did not consistently alter cytokine responses in PBMC or mucosal assays compared to media controls. These findings suggest that Sb's anti-inflammatory effects are primarily mediated through its modulation of the gut microbial community, supporting a microbiota-metabolite-immune axis that may ameliorate AMC-associated host inflammation.

While the inclusion of both static and, in particular, dynamic models strengthens the reproducibility of our microbiome findings and enables longer-term assessment of microbial recovery and resistome dynamics, these *in vitro* systems still lack key host physiological features, such as epithelial barriers, immune signaling, and spatial compartmentalization. Likewise, *ex vivo* immune assays, although translationally informative, cannot fully capture the complexity of *in vivo* immune regulation. Donor-specific variability observed across MiPro and SHIME^*®*^ experiments further underscores the heterogeneity of individual microbiome responses. These limitations should be considered when interpreting the results, and future studies incorporating more physiologically integrated host–microbiome models and larger donor cohorts will be essential to confirm and extend these findings.

In summary, this study demonstrates that *Saccharomyces boulardii* CNCM I-745 alleviates AMC-associated dysbiosis and inflammation through direct gut microbiome modulation. Using *in vitro* microbiota models and *ex vivo* co-culture systems, we show that Sb supplementation stabilizes microbial biomass, stimulates anti-inflammatory metabolites production, and mitigates antibiotic-induced pro-inflammatory effects during antibiotic treatment, particularly under AMC exposure. Our findings advance the understanding of probiotic-antibiotic-gut microbiome interactions, thereby guiding future optimization of microbiome-targeted adjuvant therapies.

## Supplementary Material

Supplementary material **Figure S1.** Growth and metabolic activity of Sb under anaerobic in vitro conditions. a Viable cell counts (Log10 CFU) of Sb at 0 and 24 h after anaerobic monoculture incubation in MiPro and SHIME® media, determined by plate counting on Sabouraud dextrose agar supplemented with 50 mg/mL chloramphenicol. b Metabolite concentrations in supernatants from 2 mg/mL (Sb+) or 4 mg/mL (Sb++) Sb monocultures after 24 h anaerobic incubation in MiPro medium. c Metabolite concentrations in supernatants from 400 mg Sb monocultures after 24 h anaerobic incubation in 200 mL of SHIME® medium. Quantification of SCFAs, tryptophan metabolites, and primary bile acids was performed using targeted LC-MS/MS. MiPro and SHIME® media served as controls.**Figure S2.** Sb supplementation has no impacts on antibiotic-induced changes in taxonomic composition in the MiPro model. a Alpha diversity of Shannon and Chao1 indices. Each color represents one stool donor in MiPro. Levels of significance were determined using paired one-way ANOVA followed by Bonferroni's post hoc test. *p < 0.05; **p < 0.01. b Beta diversity assessed by principal coordinate analysis with Bray–Curtis dissimilarity matrix of all samples at the genus level. c,d Relative abundance of microbiota at the phylum and genus levels. Data were expressed as the mean ± standard error of the mean for each taxon. e Coefficient from MaAsLin2 analysis representing the taxa with significant differences across different comparisons. Only taxa with a prevalence more than 49.9% and a q-value below 0.1 were shown.**Figure S3.** Changes in the qPCR-quantified population of specific taxa in the MiPro model. a Bacteroidota. b Bacillota. c Enterobacteriaceae. Each color represents one stool donor in MiPro. Levels of significance (a-c) were determined using paired one-way ANOVA followed by Bonferroni's post hoc test. *p < 0.05; ***p < 0.001; ****p < 0.0001.**Figure** **S4.** Significant differences in the level of metabolites across treatment groups. **a** Levels of individual SCFA. b Levels of individual tryptophan metabolite (ILA: indole-3-lactic acid; IPA: indole-3-propionic acid; I3A: indole-3-aldehyde). Each color (a,b) represents one stool donor in MiPro. Levels of significance (a,b) were determined using paired one-way ANOVA followed by Bonferroni's post hoc test. *p < 0.05; **p < 0.01; ***p < 0.001.Figure S5. A high concentration of AMC obscures Sb's protective effects on the gut microbiome in the MiPro model. **a** Changes in bacterial loads. **b** Changes in microbial production of SCFAs. A high concentration of AMC (0.5 mg/mL), together with 2 mg/mL of lyophilized Sb, was applied in the static MiPro model inoculated with 5 individual donors. Each color (a,b) represents one stool donor in MiPro. Levels of significance (a,b) were determined using paired one-way ANOVA followed by Bonferroni's post hoc test. *p < 0.05; **p < 0.01; ****p < 0.0001.**Figure S6.** Individual donor cytokine profiles from PBMCs exposed to microbiota-conditioned media from MiPro. a Cytokine levels, scaled by Z-scores, from individual PBMC donors following stimulation with PBS, LPS (100 ng/mL), MiPro® medium, or Sb (4 mg/ml; 24 h incubation) monoculture supernatant. b Cytokine levels, scaled by Z-scores, from individual PBMC donor following stimulation with MiPro supernatants. Each column displays average data from 8 stool donors in MiPro. The color in the heatmap (a,b) represents high (orange) or low (blue) cytokine levels.**Figure** **S7.** Sequences annotated to the probiotic Sb induce bias in functional profiling. a Beta diversity of microbiome functional MetaCyc pathways incorporating genome of the probiotic Sb. b Beta diversity of microbiome functional MetaCyc pathways after filtering out the Sb genome. Principal coordinate analysis was performed using centered log-ratio (CLR)-transformed relative abundance matrix and Aitchison distance. Each dot represents one donor in SHIME®. Levels of significance were determined using PERMANOVA and indicated in each plot.**Figure** **S8.** Sb supplementation has no marked impacts on AMC-induced alterations in taxonomic composition in the SHIME® model. a Alpha diversity of Shannon and Chao1 indices. Each color represents one stool donor in SHIME®. Levels of significance were determined using paired two-way ANOVA with Bonferroni's post hoc test. *p < 0.05; **p < 0.01; ***p < 0.001; ****p < 0.0001. b Beta diversity assessed by principal coordinate analysis using centered log-ratio (CLR)-transformed relative abundance matrix at the species level and Aitchison distance. Levels of significance were determined using PERMANOVA and indicated in each plot. Each dot represents one donor in SHIME® with Donor ID indicated. c,d Relative abundance of microbiota at the phylum and genus levels. Data were expressed as the mean ± standard error of the mean for each taxon. e Relative abundance of the top 20 most abundant species per donor.**Figure** **S9.** Changes in the qPCR-quantified population of specific taxa in the SHIME® model throughout the experiment and the area under the curve. a Bacteroidota. b Bacillota. c Enterobacteriaceae. Each color (a-c) represents one stool donor in SHIME®. Levels of significance (a-c) were determined using paired two-tailed Wilcoxon test. *p < 0.05.**Figure** **S10.** Sb supplementation mitigates AMC-perturbed microbiome functional dysbiosis in the SHIME® model. a Relative abundance of top 20 most abundant MetaCyc pathways per donor. b Heatmap visualization of coefficient from MaAsLin analysis representing MetaCyc pathways with significant differences between with Sb supplementation (+Sb) and without Sb supplementation (−Sb) at Day 7. Only MetaCyc pathways with a prevalence more than 49.9% and a q-value below 0.25 were shown.**Figure** **S11.** Sb supplementation mitigates AMC-induced enrichment of Enterobacteriaceae in the SHIME® model. a Spearman correlation between relative abundances of Enterobacteriaceae and antibiotic resistance genes (ARGs) targeting AMC. b Relative abundance of Enterobacteriaceae. Each color (b) represents one stool donor in SHIME®. Levels of significance (b) were determined using paired two-way ANOVA with Bonferroni's post hoc test. *p < 0.05; **p < 0.01; ***p < 0.001.**Figure** **S12.** Individual donor cytokine profiles from PBMCs exposed to microbiota-conditioned media from SHIME®. a Cytokine levels, scaled by Z-scores, from individual PBMC donors following stimulation with PBS, LPS (100 ng/mL), SHIME® medium, or Sb (400 mg in 200 mL medium; 24 h incubation) monoculture supernatant. b Cytokine levels, scaled by Z-scores, from individual PBMC donor following stimulation with SHIME® supernatants. Each column displays average data from six stool donors in SHIME®. The color in the heatmap (a,b) represents high (orange) or low (blue) cytokine levels.**Figure** **S13.** Individual donor cytokine profiles from intestinal mucosal explants exposed to microbiota-conditioned media from SHIME®. a Cytokine levels, scaled by Z-scores, from individual PBMC donors following stimulation with PBS, SHIME® medium, or Sb (400 mg in 200 mL medium; 24 h incubation) monoculture supernatant. b Cytokine levels, scaled by Z-scores, from individual mucosal explant following stimulation with SHIME® supernatants. Each column displays data from one stool donor in SHIME®. The color in the heatmap (a,b) represents high (orange) or low (blue) cytokine levels.

## Data Availability

The sequencing data have been deposited on European Nucleotide Archive (accession number PRJEB87608).

## References

[1] Ianiro G, Tilg H, Gasbarrini A. Antibiotics as deep modulators of gut microbiota: between good and evil. Gut. 2016;65:1906–1915. doi: 10.1136/gutjnl-2016-312297. 27531828

[2] Fan Y, Pedersen O. Gut microbiota in human metabolic health and disease. Nat Rev Microbiol. 2021;19:55–71. doi: 10.1038/s41579-020-0433-9. 32887946

[3] Ferrer M, Martins dos Santos VA, Ott SJ, Moya A. Gut microbiota disturbance during antibiotic therapy: a multi-omic approach. Gut Microbes. 2014;5:64–70. doi: 10.4161/gmic.27128. 24418972 PMC4049940

[4] Anthony WE, Wang B, Sukhum KV, D'Souza AW, Hink T, Cass C, Seiler S, Reske KA, Coon C, Dubberke ER, et al. Acute and persistent effects of commonly used antibiotics on the gut microbiome and resistome in healthy adults. Cell Rep. 2022;39:110649. doi: 10.1016/j.celrep.2022.110649. 35417701 PMC9066705

[5] Fenneman AC, Weidner M, Chen LA, Nieuwdorp M, Blaser MJ. Antibiotics in the pathogenesis of diabetes and inflammatory diseases of the gastrointestinal tract. Nat Rev Gastroenterol Hepatol. 2023;20:81–100. doi: 10.1038/s41575-022-00685-9. 36258032 PMC9898198

[6] Szajewska H, Scott KP, de Meij T, Forslund-Startceva SK, Knight R, Koren O, Little P, Johnston BC, Łukasik J, Suez J, et al. Antibiotic-perturbed microbiota and the role of probiotics. Nat Rev Gastroenterol Hepatol. 2025;22:155–172. doi: 10.1038/s41575-024-01023-x. 39663462

[7] Waitzberg D, Guarner F, Hojsak I, Ianiro G, Polk DB, Sokol H. Can the evidence-based use of probiotics (notably saccharomyces boulardii CNCM I-745 and lactobacillus rhamnosus GG) mitigate the clinical effects of antibiotic-associated dysbiosis?. Adv Ther. 2024;41:901–914. doi: 10.1007/s12325-024-02783-3. 38286962 PMC10879266

[8] Chen K, Zhu Y, Zhang Y, Hamza T, Yu H, Saint Fleur A, Galen J, Yang Z, Feng H. A probiotic yeast-based immunotherapy against Clostridioides difficile infection. Sci Transl Med. 2020;12(567). doi: 10.1126/scitranslmed.aax4905. PMC7692727 33115949

[9] Kotowska M, Albrecht P, Szajewska H. Saccharomyces boulardii in the prevention of antibiotic-associated diarrhoea in children: a randomized double-blind placebo-controlled trial. Aliment Pharmacol Ther. 2005;21:583–590. doi: 10.1111/j.1365-2036.2005.02356.x. 15740542

[10] Surawicz CM, Elmer GW, Speelman P, McFarland LV, Chinn J, Van Belle G. Prevention of antibiotic-associated diarrhea by Saccharomyces boulardii: a prospective study. Gastroenterology. 1989;96:981–988. doi: 10.1016/0016-5085(89)91613-2. 2494098

[11] Wombwell E, Patterson ME, Bransteitter B, Gillen LR. The effect of saccharomyces boulardii primary prevention on risk of hospital-onset clostridioides difficile infection in hospitalized patients administered antibiotics frequently associated with c. difficile infection. Clin Infect Dis. 2021;73:e2512–e2518. doi: 10.1093/cid/ciaa808. 32575126

[12] Cardenas PA, Cárdenas PA, Garcés D, Prado-Vivar B, Flores N, Fornasini M, Cohen H, Salvador I, Cargua O, Baldeón ME. Effect of Saccharomyces boulardii CNCM I-745 as complementary treatment of Helicobacter pylori infection on gut microbiome. Eur J Clin Microbiol Infect Dis. 2020;39:1365–1372. doi: 10.1007/s10096-020-03854-3. 32125555

[13] Kabbani TA, Pallav K, Dowd SE, Villafuerte-Galvez J, Vanga RR, Castillo NE, Hansen J, Dennis M, Leffler DA, Kelly CP. Prospective randomized controlled study on the effects of Saccharomyces boulardii CNCM I-745 and amoxicillin-clavulanate or the combination on the gut microbiota of healthy volunteers. Gut Microbes. 2017;8:17–32. doi: 10.1080/19490976.2016.1267890. 27973989 PMC5341914

[14] Spatz M, Wang Y, Lapiere A, Da Costa G, Michaudel C, Danne C, Michel M, Langella P, Sokol H, Richard ML. Saccharomyces boulardii CNCM I-745 supplementation during and after antibiotic treatment positively influences the bacterial gut microbiota. Front Med (Lausanne). 2023;10:1087715. doi: 10.3389/fmed.2023.1087715. 37601783 PMC10436532

[15] Heavey MK, Hazelton A, Wang Y, Garner M, Anselmo AC, Arthur JC, Nguyen J. Targeted delivery of the probiotic Saccharomyces boulardii to the extracellular matrix enhances gut residence time and recovery in murine colitis. Nat Commun. 2024;15:3784. doi: 10.1038/s41467-024-48128-0. 38710716 PMC11074276

[16] Hoffmann TW, Pham H, Bridonneau C, Aubry C, Lamas B, Martin-Gallausiaux C, Moroldo M, Rainteau D, Lapaque N, Six A, et al. Microorganisms linked to inflammatory bowel disease-associated dysbiosis differentially impact host physiology in gnotobiotic mice. ISME J. 2016;10:460–477. doi: 10.1038/ismej.2015.127. 26218241 PMC4737937

[17] Aranda-Diaz A, Aranda-Díaz A, Ng KM, Thomsen T, Real-Ramírez I, Dahan D, Dittmar S, Gonzalez CG, Chavez T, Vasquez KS, et al. Establishment and characterization of stable, diverse, fecal-derived in vitro microbial communities that model the intestinal microbiota. Cell Host Microbe. 2022;30:260–272. doi: 10.1016/j.chom.2021.12.008. 35051349 PMC9082339

[18] Li L, Abou-Samra E, Ning Z, Zhang X, Mayne J, Wang J, Cheng K, Walker K, Stintzi A, Figeys D. An in vitro model maintaining taxon-specific functional activities of the gut microbiome. Nat Commun. 2019;10:4146. doi: 10.1038/s41467-019-12087-8. 31515476 PMC6742639

[19] Rytter H, Naimi S, Wu G, Lewis J, Duquesnoy M, Vigué L, Tenaillon O, Belda E, Vazquez-Gomez M, Touly N, et al. In vitro microbiota model recapitulates and predicts individualised sensitivity to dietary emulsifier. Gut. 2025;74:761–774. doi: 10.1136/gutjnl-2024-333925. 39870396 PMC12013555

[20] Wu L, Wang X, Tao Z, Zuo W, Zeng Y, Liu Y, Dai L. Data-driven prediction of colonization outcomes for complex microbial communities. Nat Commun. 2024;15:2406. doi: 10.1038/s41467-024-46766-y. 38493186 PMC10944475

[21] Van de Wiele T, Van den Abbeele P, Ossieur W, Possemiers S, Marzorati M. in The Impact of food bioactives on health: in vitro and ex vivo models. In K Verhoeckx (Ed.), 2015. pp. 305–315 Cham (CH): Springer. 29787039

[22] Anjou C, Royer M, Bertrand É, Bredon M, Le Bris J, Salgueiro IA, Caulat LC, Dupuy B, Barbut F, Morvan C, et al. Adaptation mechanisms of Clostridioides difficile to auranofin and its impact on human gut microbiota. NPJ Biofilms Microbiomes. 2024;10:86. doi: 10.1038/s41522-024-00551-3. 39284817 PMC11405772

[23] Minnebo Y, Delbaere K, Goethals V, Raes J, Van de Wiele T, De Paepe K. Gut microbiota response to in vitro transit time variation is mediated by microbial growth rates, nutrient use efficiency and adaptation to in vivo transit time. Microbiome. 2023;11:240. doi: 10.1186/s40168-023-01691-y. 37926855 PMC10626715

[24] Sender R, Fuchs S, Milo R. Revised estimates for the number of human and bacteria cells in the body. PLoS Biol. 2016;14:e1002533. doi: 10.1371/journal.pbio.1002533. 27541692 PMC4991899

[25] Huang Z, Boekhorst J, Fogliano V, Capuano E, Wells JM. Impact of high-fiber or high-protein diet on the capacity of human gut microbiota to produce tryptophan catabolites. J Agric Food Chem. 2023;71:6956–6966. doi: 10.1021/acs.jafc.2c08953. 37126824 PMC10176579

[26] Lamas B, Richard ML, Leducq V, Pham H, Michel M, Da Costa G, Bridonneau C, Jegou S, Hoffmann TW, Natividad JM, et al. CARD9 impacts colitis by altering gut microbiota metabolism of tryptophan into aryl hydrocarbon receptor ligands. Nat Med. 2016;22:598–605. doi: 10.1038/nm.4102. 27158904 PMC5087285

[27] Bolyen E, Rideout JR, Dillon MR, Bokulich NA, Abnet CC, Al-Ghalith GA, Alexander H, Alm EJ, Arumugam M, Asnicar F, et al. Reproducible, interactive, scalable and extensible microbiome data science using QIIME 2. Nat Biotechnol. 2019;37:852–857. doi: 10.1038/s41587-019-0209-9. 31341288 PMC7015180

[28] Callahan BJ, McMurdie PJ, Rosen MJ, Han AW, Johnson AJA, Holmes SP. DADA2: High-resolution sample inference from Illumina amplicon data. Nat Methods. 2016;13:581–583. doi: 10.1038/nmeth.3869. 27214047 PMC4927377

[29] Quast C, Pruesse E, Yilmaz P, Gerken J, Schweer T, Yarza P, Peplies J, Glöckner FO. The SILVA ribosomal RNA gene database project: improved data processing and web-based tools. Nucleic Acids Res. 2013;41:D590–596. doi: 10.1093/nar/gks1219. 23193283 PMC3531112

[30] McMurdie PJ, Holmes S. phyloseq: an R package for reproducible interactive analysis and graphics of microbiome census data. PLoS One. 2013;8:e61217. doi: 10.1371/journal.pone.0061217. 23630581 PMC3632530

[31] Mallick H, Rahnavard A, McIver LJ, Ma S, Zhang Y, Nguyen LH, Tickle TL, Weingart G, Ren B, Schwager EH, et al. Multivariable association discovery in population-scale meta-omics studies. PLoS Comput Biol. 2021;17:e1009442. doi: 10.1371/journal.pcbi.1009442. 34784344 PMC8714082

[32] Segata N, Waldron L, Ballarini A, Narasimhan V, Jousson O, Huttenhower C. Metagenomic microbial community profiling using unique clade-specific marker genes. Nat Methods. 2012;9:811–814. doi: 10.1038/nmeth.2066. 22688413 PMC3443552

[33] Franzosa EA, McIver LJ, Rahnavard G, Thompson LR, Schirmer M, Weingart G, Lipson KS, Knight R, Caporaso JG, Segata N, et al. Species-level functional profiling of metagenomes and metatranscriptomes. Nat Methods. 2018;15:962–968. doi: 10.1038/s41592-018-0176-y. 30377376 PMC6235447

[34] Bortolaia V, Kaas RS, Ruppe E, Roberts MC, Schwarz S, Cattoir V, Philippon A, Allesoe RL, Rebelo AR, Florensa AF, et al. ResFinder 4.0 for predictions of phenotypes from genotypes. J Antimicrob Chemother. 2020;75:3491–3500. doi: 10.1093/jac/dkaa345. 32780112 PMC7662176

[35] Gschwind R, Ugarcina Perovic S, Weiss M, Petitjean M, Lao J, Coelho LP, Ruppé E. ResFinderFG v2.0: a database of antibiotic resistance genes obtained by functional metagenomics. Nucleic Acids Res. 2023;51:W493–W500. doi: 10.1093/nar/gkad384. 37207327 PMC10320180

[36] Yin X, Zheng X, Li L, Zhang A, Jiang X. ARGs-OAP v3.0: antibiotic-resistance gene database curation and analysis pipeline optimization. Engineering. 2023;27:234–241. doi: 10.1016/j.eng.2022.10.011.

[37] Nishijima S, Stankevic E, Aasmets O, Schmidt TS, Nagata N, Keller MI, Ferretti P, Juel HB, Fullam A, Robbani SM, et al. Fecal microbial load is a major determinant of gut microbiome variation and a confounder for disease associations. Cell. 2025;188:222–236. doi: 10.1016/j.cell.2024.10.022. 39541968

[38] Alarcan H, Chaumond R, Emond P, Benz-De Bretagne I, Lefèvre A, Bakkouche S, Veyrat-Durebex C, Vourc'h P, Andres C, Corcia P, et al. Some CSF kynurenine pathway intermediates associated with disease evolution in amyotrophic lateral sclerosis. Biomolecules. 2021;11:691. doi: 10.3390/biom11050691. 34063031 PMC8147980

[39] Mahdi T, Desmons A, Krasniqi P, Lacorte J, Kapel N, Lamazière A, Fourati S, Eguether T. Effect of stool sampling on a routine clinical method for the quantification of six short chain fatty acids in stool using gas chromatography-mass spectrometry. Microorganisms. 2024;12:828. doi: 10.3390/microorganisms12040828. 38674773 PMC11052040

[40] Monteiro J, Lefèvre A, Dufour-Rainfray D, Oury A, Chicheri G, Galineau L, Blasco H, Nadal-Desbarats L, Emond P. Multi-compartment SCFA quantification in human. Am J Anal Chem. 2024;15:177–200. doi: 10.4236/ajac.2024.156012.

[41] Bottery MJ, Matthews JL, Wood AJ, Johansen HK, Pitchford JW, Friman V. Inter-species interactions alter antibiotic efficacy in bacterial communities. ISME J. 2022;16:812–821. doi: 10.1038/s41396-021-01130-6. 34628478 PMC8857223

[42] Isaac S, Scher JU, Djukovic A, Jiménez N, Littman DR, Abramson SB, Pamer EG, Ubeda C. Short- and long-term effects of oral vancomycin on the human intestinal microbiota. J Antimicrob Chemother. 2017;72:128–136. doi: 10.1093/jac/dkw383. 27707993 PMC5161046

[43] Vandeputte D, Kathagen G, D'hoe K, Vieira-Silva S, Valles-Colomer M, Sabino J, Wang J, Tito RY, De Commer L, Darzi Y, et al. Quantitative microbiome profiling links gut community variation to microbial load. Natur. 2017;551:507–511. doi: 10.1038/nature24460. 29143816

[44] Lee JH, Lee J. Indole as an intercellular signal in microbial communities. FEMS Microbiol Rev. 2010;34:426–444. doi: 10.1111/j.1574-6976.2009.00204.x. 20070374

[45] Kelly CP, Chong Nguyen C, Palmieri LJ, Pallav K, Dowd SE, Humbert L, Seksik P, Bado A, Coffin B, Rainteau D, et al. Saccharomyces boulardii CNCM I-745 modulates the fecal bile acids metabolism during antimicrobial therapy in healthy volunteers. Front Microbiol. 2019;10:336. doi: 10.3389/fmicb.2019.00336. 30881353 PMC6407479

[46] Guo P, Zhang K, Ma X, He P. Clostridium species as probiotics: potentials and challenges. J Anim Sci Biotechnol. 2020;11:24. doi: 10.1186/s40104-019-0402-1. 32099648 PMC7031906

[47] Le Roy T, Moens de Hase E, Van Hul M, Paquot A, Pelicaen R, Régnier M, Depommier C, Druart C, Everard A, Maiter D, et al. Dysosmobacter welbionis is a newly isolated human commensal bacterium preventing diet-induced obesity and metabolic disorders in mice. Gut. 2022;71:534–543. doi: 10.1136/gutjnl-2020-323778. 34108237 PMC8862106

[48] Sun Y, Nie Q, Zhang S, He H, Zuo S, Chen C, Yang J, Hu J, Li S, Cheng J, et al. Parabacteroides distasonis ameliorates insulin resistance via activation of intestinal GPR109a. Nat Commun. 2023;14:7740. doi: 10.1038/s41467-023-43622-3. 38007572 PMC10676405

[49] Yan Y, Lei Y, Qu Y, Fan Z, Zhang T, Xu Y, Du Q, Brugger D, Chen Y. Bacteroides uniformis-induced perturbations in colonic microbiota and bile acid levels inhibit TH17 differentiation and ameliorate colitis developments. NPJ Biofilms Microbiomes. 2023;9:56. doi: 10.1038/s41522-023-00420-5. 37580334 PMC10425470

[50] Parker BJ, Wearsch PA, Veloo ACM, Rodriguez-Palacios A. The genus alistipes: gut bacteria with emerging implications to inflammation, cancer, and mental health. Front Immunol. 2020;11:906. doi: 10.3389/fimmu.2020.00906. 32582143 PMC7296073

[51] Tian L, Wang X, Wu A, Fan Y, Friedman J, Dahlin A, Waldor MK, Weinstock GM, Weiss ST, Liu Y. Deciphering functional redundancy in the human microbiome. Nat Commun. 2020;11:6217. doi: 10.1038/s41467-020-19940-1. 33277504 PMC7719190

[52] Mann ER, Lam YK, Uhlig HH. Short-chain fatty acids: linking diet, the microbiome and immunity. Nat Rev Immunol. 2024;24:577–595. doi: 10.1038/s41577-024-01014-8. 38565643

[53] Lavelle A, Sokol H. Gut microbiota-derived metabolites as key actors in inflammatory bowel disease. Nat Rev Gastroenterol Hepatol. 2020;17:223–237. doi: 10.1038/s41575-019-0258-z. 32076145

[54] Fishbein SRS, Mahmud B, Dantas G. Antibiotic perturbations to the gut microbiome. Nat Rev Microbiol. 2023;21:772–788. doi: 10.1038/s41579-023-00933-y. 37491458 PMC12087466

[55] More MI, Swidsinski A. Saccharomyces boulardii CNCM I-745 supports regeneration of the intestinal microbiota after diarrheic dysbiosis - a review. Clin Exp Gastroenterol. 2015;8:237–255. doi: 10.2147/CEG.S85574. 26316791 PMC4542552

[56] Selig DJ, DeLuca JP, Li Q, Lin H, Nguyen K, Scott SM, Sousa JC, Vuong CT, Xie LH, Livezey JR. Saccharomyces boulardii CNCM I-745 probiotic does not alter the pharmacokinetics of amoxicillin. Drug Metab Pers Ther. 2020;35. doi: 10.1515/dmpt-2019-0032. 32134728

[57] Wilde J, Slack E, Foster KR. Host control of the microbiome: mechanisms, evolution, and disease. Sci. 2024;385:eadi3338. doi: 10.1126/science.adi3338. 39024451

[58] Pereira FC, Ge X, Kristensen JM, Kirkegaard RH, Maritsch K, Szamosvári D, Imminger S, Seki D, Shazzad JB, Zhu Y, et al. The Parkinson's disease drug entacapone disrupts gut microbiome homoeostasis via iron sequestration. Nat Microbiol. 2024;9:3165–3183. doi: 10.1038/s41564-024-01853-0. 39572788 PMC11602724

[59] Tito RY, Verbandt S, Aguirre Vazquez M, Lahti L, Verspecht C, Lloréns-Rico V, Vieira-Silva S, Arts J, Falony G, Dekker E, et al. Microbiome confounders and quantitative profiling challenge predicted microbial targets in colorectal cancer development. Nat Med. 2024;30:1339–1348. doi: 10.1038/s41591-024-02963-2. 38689063 PMC11108775

[60] Vieira-Silva S, Sabino J, Valles-Colomer M, Falony G, Kathagen G, Caenepeel C, Cleynen I, van der Merwe S, Vermeire S, Raes J. Quantitative microbiome profiling disentangles inflammation- and bile duct obstruction-associated microbiota alterations across PSC/IBD diagnoses. Nat Microbiol. 2019;4:1826–1831. doi: 10.1038/s41564-019-0483-9. 31209308

[61] Argentini C, Mancabelli L, Alessandri G, Tarracchini C, Barbetti M, Carnevali L, Longhi G, Viappiani A, Anzalone R, Milani C, et al. Exploring the ecological effects of naturally antibiotic-insensitive bifidobacteria in the recovery of the resilience of the gut microbiota during and after antibiotic treatment. Appl Environ Microbiol. 2022;88:e0052222. doi: 10.1128/aem.00522-22. 35652662 PMC9238419

[62] Nogacka AM, Saturio S, Alvarado-Jasso GM, Salazar N, de los Reyes Gavilán CG, Martínez-Faedo C, Suarez A, Wang R, Miyazawa K, Harata G, et al. Probiotic-induced modulation of microbiota composition and antibiotic resistance genes load, an in vitro assessment. Int J Mol Sci. 2024;25:1003. doi: 10.3390/ijms25021003. 38256076 PMC10816173

[63] Tierney BT, Van den Abbeele P, Al-Ghalith GA, Verstrepen L, Ghyselinck J, Calatayud M, Marzorati M, Gadir AA, Daisley B, Reid G, et al. Capacity of a microbial synbiotic to rescue the in vitro metabolic activity of the gut microbiome following perturbation with alcohol or antibiotics. Appl Environ Microbiol. 2023;89:e0188022. doi: 10.1128/aem.01880-22. 36840551 PMC10056957

[64] Louca S, Polz MF, Mazel F, Albright MBN, Huber JA, O'Connor MI, Ackermann M, Hahn AS, Srivastava DS, Crowe SA, et al. Function and functional redundancy in microbial systems. Nat Ecol Evol. 2018;2:936–943. doi: 10.1038/s41559-018-0519-1. 29662222

[65] Plaza-Diaz J, Ruiz-Ojeda FJ, Gil-Campos M, Gil A. Mechanisms of action of probiotics. Adv Nutr. 2019;10:S49–S66. doi: 10.1093/advances/nmy063. 30721959 PMC6363529

[66] Charlet R, Bortolus C, Sendid B, Jawhara S. Bacteroides thetaiotaomicron and Lactobacillus johnsonii modulate intestinal inflammation and eliminate fungi via enzymatic hydrolysis of the fungal cell wall. Sci Rep. 2020;10:11510. doi: 10.1038/s41598-020-68214-9. 32661259 PMC7359362

[67] Huang W, Cho KY, Meng D, Walker WA. The impact of indole-3-lactic acid on immature intestinal innate immunity and development: a transcriptomic analysis. Sci Rep. 2021;11:8088. doi: 10.1038/s41598-021-87353-1. 33850185 PMC8044159

[68] Meng D, Sommella E, Salviati E, Campiglia P, Ganguli K, Djebali K, Zhu W, Walker WA. Indole-3-lactic acid, a metabolite of tryptophan, secreted by Bifidobacterium longum subspecies infantis is anti-inflammatory in the immature intestine. Pediatr Res. 2020;88:209–217. doi: 10.1038/s41390-019-0740-x. 31945773 PMC7363505

[69] Perdijk O, Butler A, Macowan M, Chatzis R, Bulanda E, Grant RD, Harris NL, Wypych TP, Marsland BJ. Antibiotic-driven dysbiosis in early life disrupts indole-3-propionic acid production and exacerbates allergic airway inflammation in adulthood. Immunity. 2024;57:1939–1954. 10.1016/j.immuni.2024.06.010. 39013465

[70] Serger E, Luengo-Gutierrez L, Chadwick JS, Kong G, Zhou L, Crawford G, Danzi MC, Myridakis A, Brandis A, Bello AT, et al. The gut metabolite indole-3 propionate promotes nerve regeneration and repair. Natur. 2022;607:585–592. doi: 10.1038/s41586-022-04884-x. 35732737

[71] Natividad JM, Agus A, Planchais J, Lamas B, Jarry AC, Martin R, Michel M, Chong-Nguyen C, Roussel R, Straube M, et al. Impaired aryl hydrocarbon receptor ligand production by the gut microbiota is a key factor in metabolic syndrome. Cell Metab. 2018;28:737–749. doi: 10.1016/j.cmet.2018.07.001. 30057068

[72] Huang Z, Wells JM, Fogliano V, Capuano E. Microbial tryptophan catabolism as an actionable target via diet-microbiome interactions. Crit Rev Food Sci Nutr. 2025;65:3650–3664. doi: 10.1080/10408398.2024.2369947. 38950607

[73] Hedin KA, Mirhakkak MH, Vaaben TH, Sands C, Pedersen M, Baker A, Vazquez-Uribe R, Schäuble S, Panagiotou G, Wellejus A, et al. Saccharomyces boulardii enhances anti-inflammatory effectors and AhR activation via metabolic interactions in probiotic communities. ISME J. 2024;18(1):wrae212. doi: 10.1093/ismejo/wrae212. 39488793 PMC11631509

[74] Bradford PA. Extended-spectrum beta-lactamases in the 21st century: characterization, epidemiology, and detection of this important resistance threat. Clin Microbiol Rev. 2001;14:933–951. doi: 10.1128/CMR.14.4.933-951.2001. table of contents. 11585791 PMC89009

[75] Button JE, Cosetta CM, Reens AL, Brooker SL, Rowan-Nash AD, Lavin RC, Saur R, Zheng S, Autran CA, Lee ML, et al. Precision modulation of dysbiotic adult microbiomes with a human-milk-derived synbiotic reshapes gut microbial composition and metabolites. Cell Host Microbe. 2023;31:1523–1538. doi: 10.1016/j.chom.2023.08.004. 37657443

[76] Yang N, Ma T, Xie Y, Li Q, Zheng L, Xiao Q, Sun Z, Zuo K, Kwok L, Lu N, et al. Lactiplantibacillus plantarum P9 for chronic diarrhea in young adults: a large double-blind, randomized, placebo-controlled trial. Nat Commun. 2024;15:6823. doi: 10.1038/s41467-024-51094-2. 39122704 PMC11315937

[77] Arpaia N, Campbell C, Fan X, Dikiy S, van der Veeken J, deRoos P, Liu H, Cross JR, Pfeffer K, Coffer PJ, et al. Metabolites produced by commensal bacteria promote peripheral regulatory T-cell generation. Natur. 2013;504:451–455. doi: 10.1038/nature12726. PMC3869884 24226773

[78] Zhao ZH, Xin F, Xue Y, Hu Z, Han Y, Ma F, Zhou D, Liu X, Cui A, Gao J, et al. Indole-3-propionic acid inhibits gut dysbiosis and endotoxin leakage to attenuate steatohepatitis in rats. Exp Mol Med. 2019;51:1–14. doi: 10.1038/s12276-019-0304-5. PMC6802644 31506421

