# Supplementary material for: Saccharomyces boulardii CNCM I-745 mitigates antibiotic-induced gut microbiome functional alterations independently of the host
Source: Gut Microbes. 2025 Nov 7;17(1):2575924. doi: 10.1080/19490976.2025.2575924 (PMC12599567; doi:10.1080/19490976.2025.2575924)
Supplement: Supplementary material — Figure S1. Growth and metabolic activity of Sb under anaerobic in vitro conditions. a Viable cell counts (Log10 CFU) of Sb at 0 and 24 h after anaerobic monoculture incubation in MiPro and SHIME® media, determined by plate counting on Sabouraud dextrose agar supplemented with 50 mg/mL chloramphenicol. b Metabolite concentrations in supernatants from 2 mg/mL (Sb+) or 4 mg/mL (Sb++) Sb monocultures after 24 h anaerobic incubation in MiPro medium. c Metabolite concentrations in supernatants from 400 mg Sb monocultures after 24 h anaerobic incubation in 200 mL of SHIME® medium. Quantification of SCFAs, tryptophan metabolites, and primary bile acids was performed using targeted LC-MS/MS. MiPro and SHIME® media served as controls.Figure S2. Sb supplementation has no impacts on antibiotic-induced changes in taxonomic composition in the MiPro model. a Alpha diversity of Shannon and Chao1 indices. Each color represents one stool donor in MiPro. Levels of significance were determined using paired one-way ANOVA followed by Bonferroni's post hoc test. *p < 0.05; **p < 0.01. b Beta diversity assessed by principal coordinate analysis with Bray–Curtis dissimilarity matrix of all samples at the genus level. c,d Relative abundance of microbiota at the phylum and genus levels. Data were expressed as the mean ± standard error of the mean for each taxon. e Coefficient from MaAsLin2 analysis representing the taxa with significant differences across different comparisons. Only taxa with a prevalence more than 49.9% and a q-value below 0.1 were shown.Figure S3. Changes in the qPCR-quantified population of specific taxa in the MiPro model. a Bacteroidota. b Bacillota. c Enterobacteriaceae. Each color represents one stool donor in MiPro. Levels of significance (a-c) were determined using paired one-way ANOVA followed by Bonferroni's post hoc test. *p < 0.05; ***p < 0.001; ****p < 0.0001.Figure S4. Significant differences in the level of metabolites across treatment groups. a Levels of indiv [file KGMI_A_2575924_SM7891.pdf]

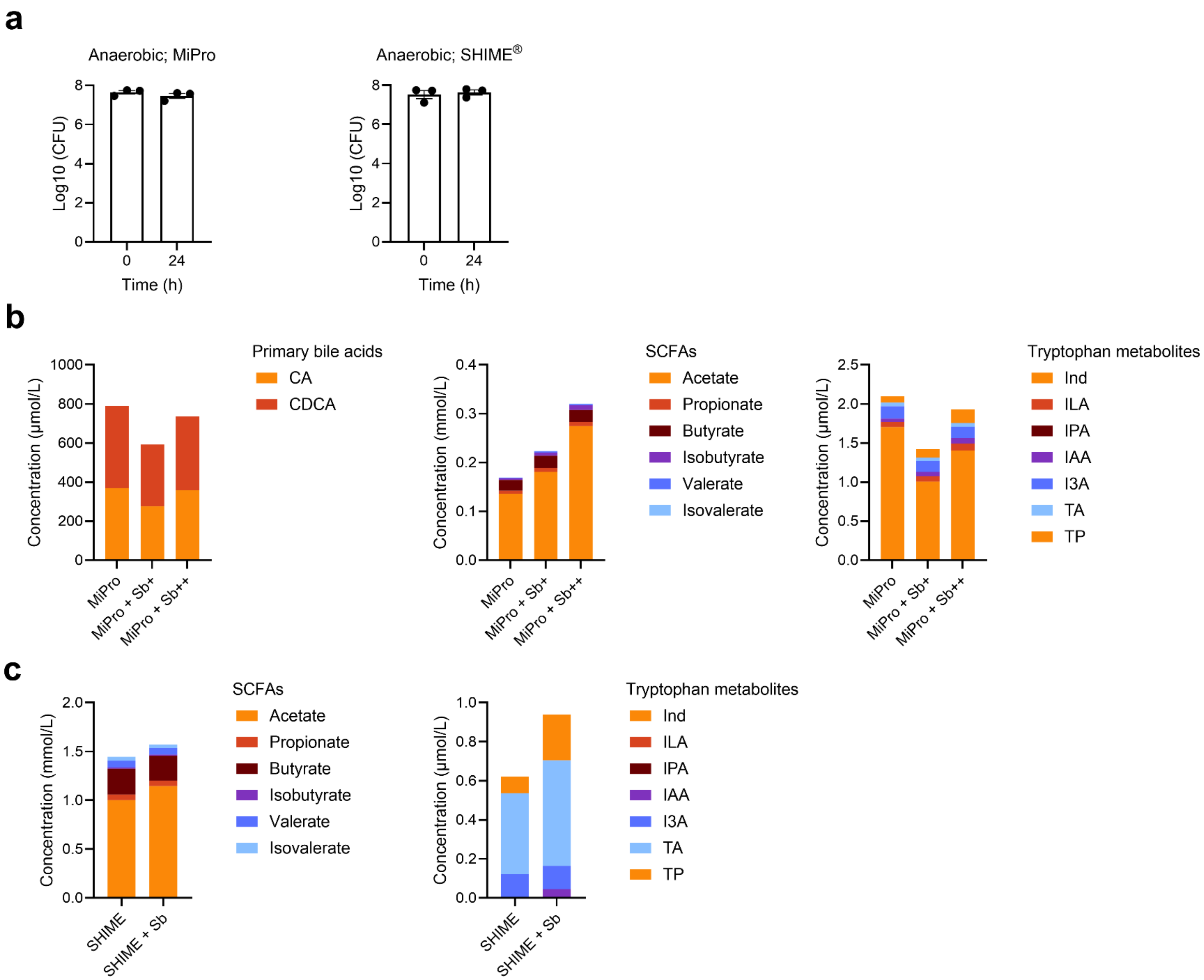
a
Anaerobic; MiPro
Anaerobic; SHIME®
Log10 (CFU)
Time (h)
0
24
b
Primary bile acids
CA
CDCA
Concentration (µmol/L)
MiPro
MiPro + Sb+
MiPro + Sb++
SCFAs
Acetate
Propionate
Butyrate
Isobutyrate
Valerate
Isovalerate
Concentration (mmol/L)
Tryptophan metabolites
Ind
ILA
IPA
IAA
I3A
TA
TP
c
SHIME
SHIME + Sb

Figure S1

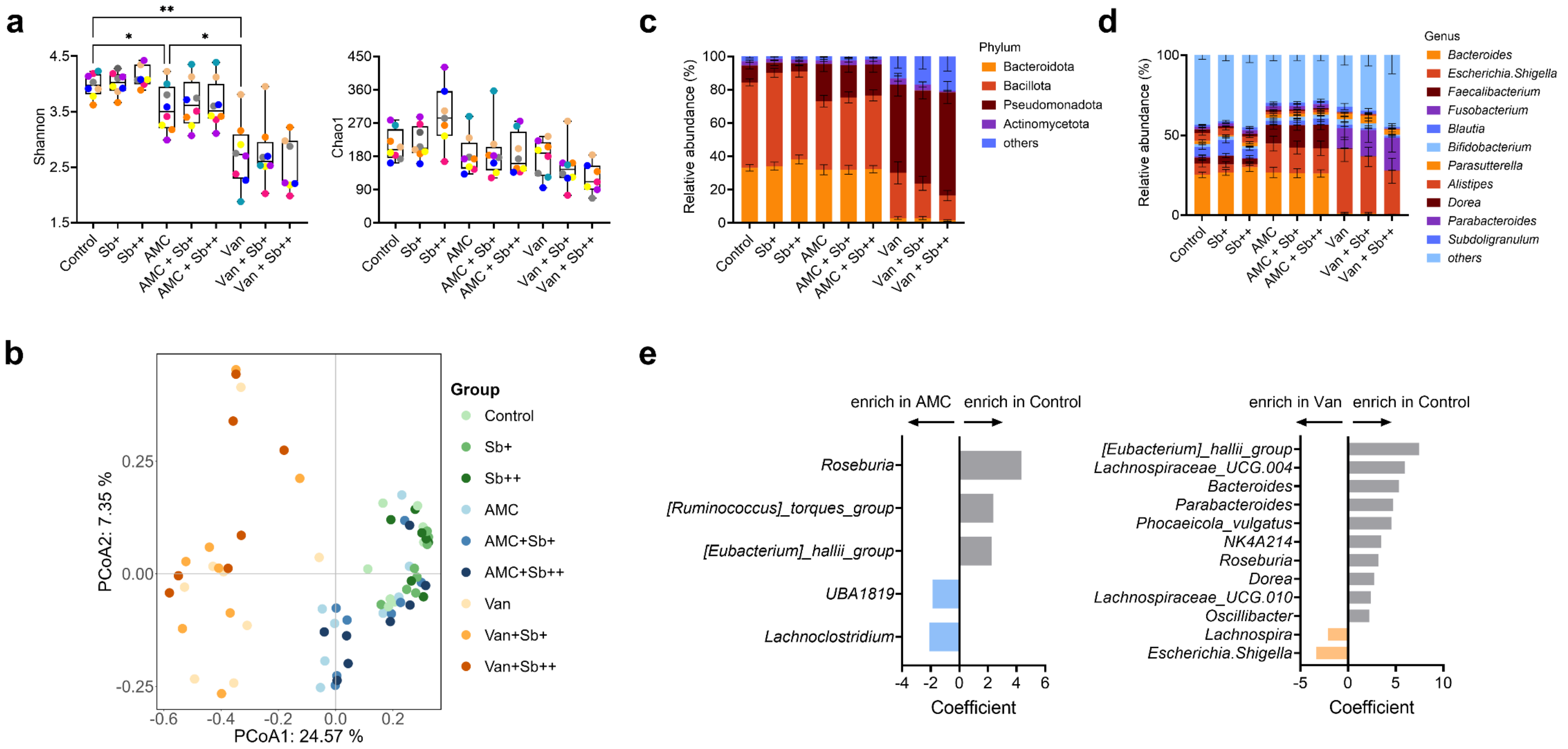
a
Shannon
Chao1
Control
Sb+
Sb++
AMC
AMC + Sb+
AMC + Sb++
Van
Van + Sb+
Van + Sb++
b
PCoA1: 24.57 %
PCoA2: 7.35 %
Group
Control
Sb+
Sb++
AMC
AMC+Sb+
AMC+Sb++
Van
Van+Sb+
Van+Sb++
c
Relative abundance (%)
Phylum
Bacteroidota
Bacillota
Pseudomonadota
Actinomycetota
others
d
Relative abundance (%)
Genus
Bacteroides
Escherichia.Shigella
Faecalibacterium
Fusobacterium
Blautia
Bifidobacterium
Parasutterella
Alistipes
Dorea
Parabacteroides
Subdoligranulum
others
e
enrich in AMC
enrich in Control
Roseburia
[Ruminococcus]_torques_group
[Eubacterium]_hallii_group
UBA1819
Lachnoclostridium
Coefficient
enrich in Van
enrich in Control
[Eubacterium]_hallii_group
Lachnospiraceae_UCG.004
Bacteroides
Parabacteroides
Phocaeicola_vulgatus
NK4A214
Roseburia
Dorea
Lachnospiraceae_UCG.010
Oscillibacter
Lachnospira
Escherichia.Shigella
Coefficient

Figure S2

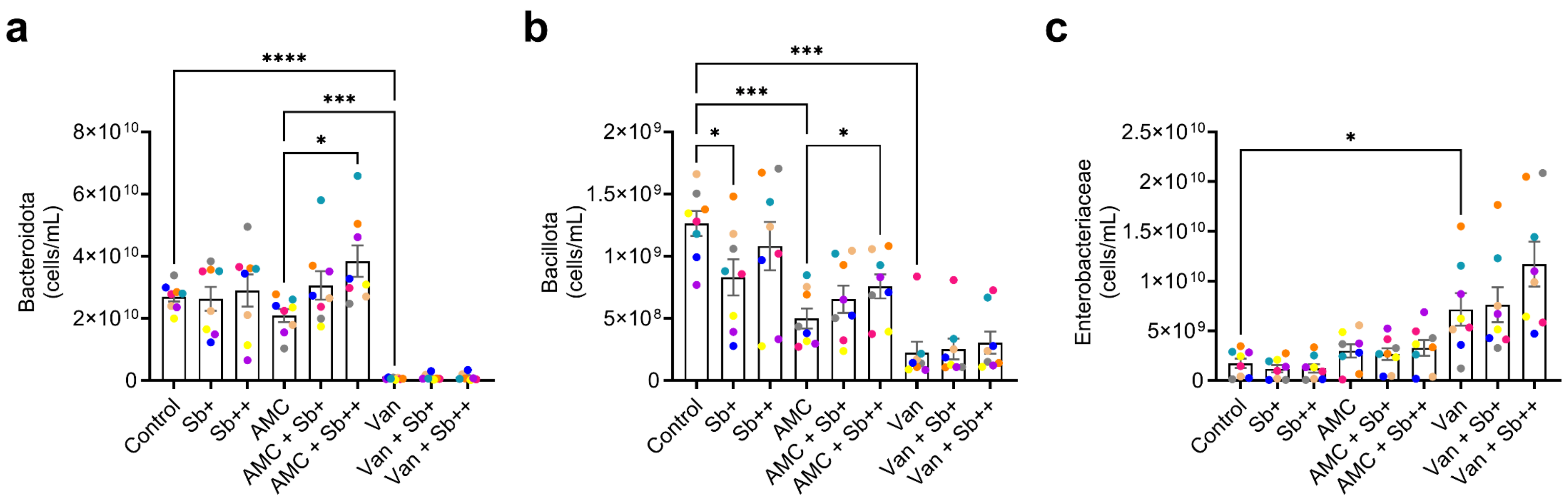
a
b
c
Bacteroidota (cells/mL)
Bacillota (cells/mL)
Enterobacteriaceae (cells/mL)
Control
Sb+
Sb++
AMC
AMC + Sb+
AMC + Sb++
Van
Van + Sb+
Van + Sb++

Figure S3

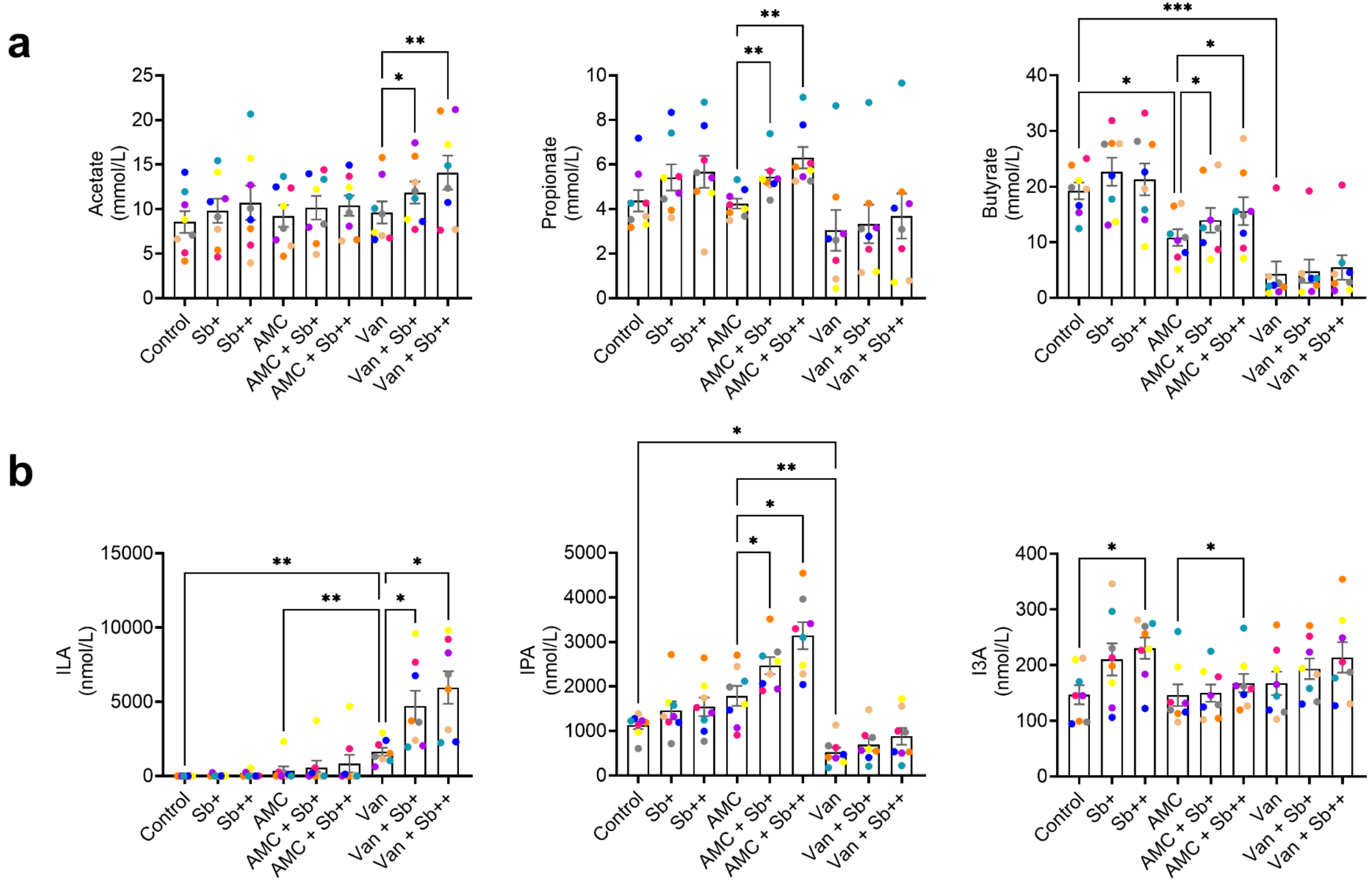

a
Acetate (mmol/L)
Propionate (mmol/L)
Butyrate (mmol/L)
b
ILA (nmol/L)
IPA (nmol/L)
I3A (nmol/L)
Control
Sb+
Sb++
AMC
AMC + Sb+
AMC + Sb++
Van
Van + Sb+
Van + Sb++

Figure S4

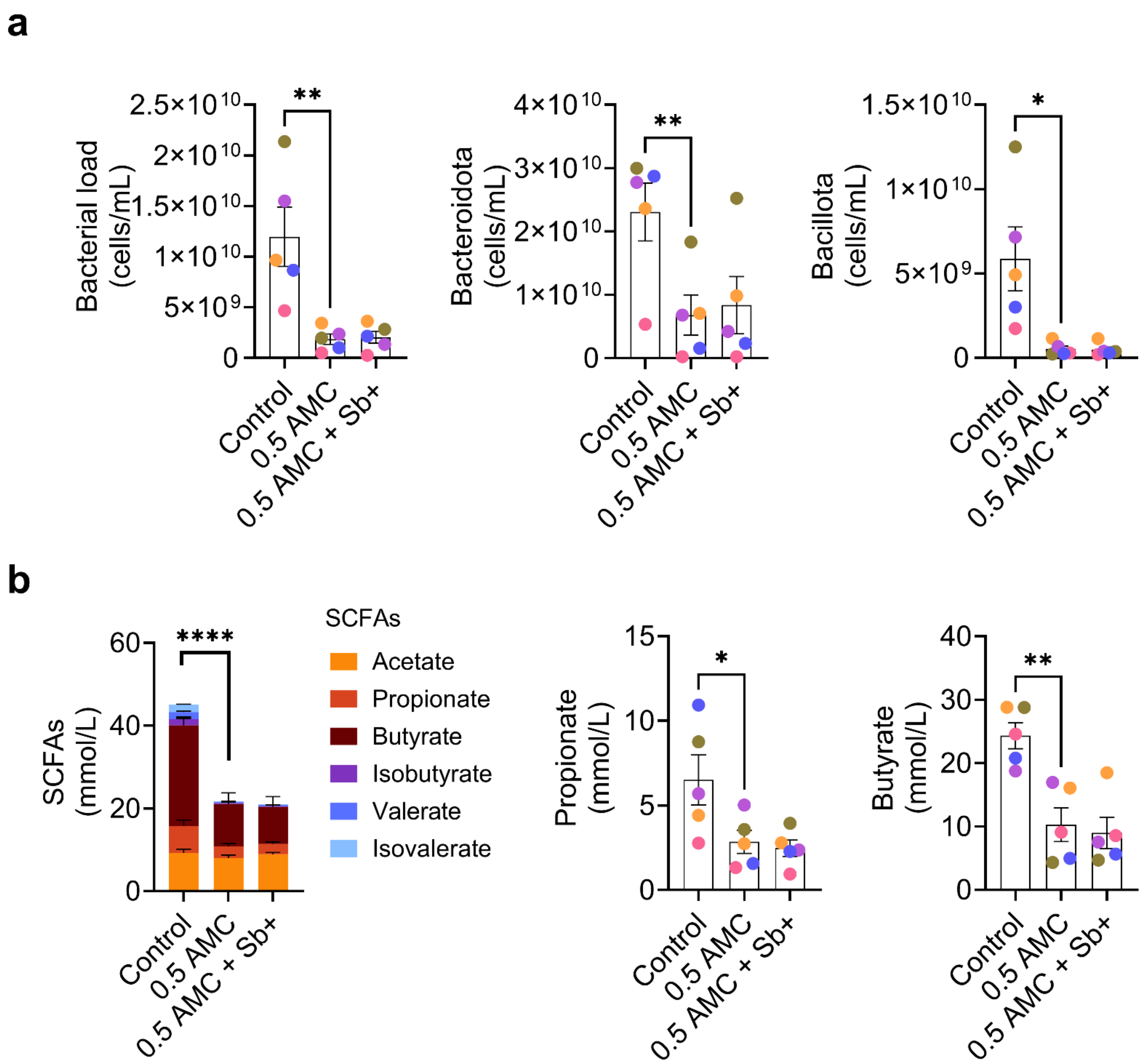

a
Bacterial load (cells/mL)
2.5×10^10
2×10^10
1.5×10^10
1×10^10
5×10^9
0
**
Control
0.5 AMC
0.5 AMC + Sb+
Bacteroidota (cells/mL)
4×10^10
3×10^10
2×10^10
1×10^10
**
Bacillota (cells/mL)
1.5×10^10
1×10^10
5×10^9
*
b
SCFAs (mmol/L)
60
40
20
****
SCFAs
Acetate
Propionate
Butyrate
Isobutyrate
Valerate
Isovalerate
Propionate (mmol/L)
15
10
5
*
Butyrate (mmol/L)
40
30
20
10
**

Figure S5

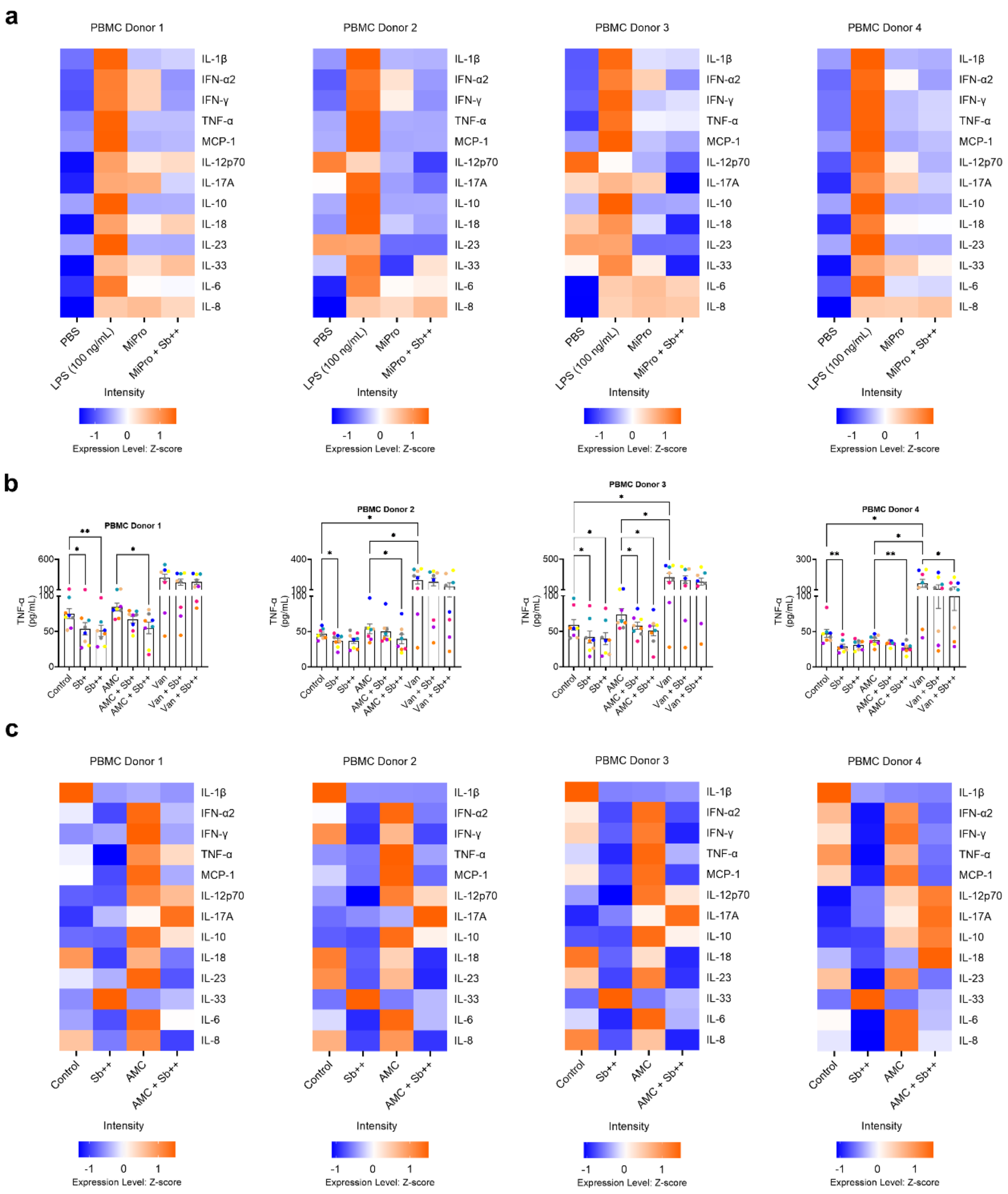
a
PBMC Donor 1
PBMC Donor 2
PBMC Donor 3
PBMC Donor 4
IL-1β
IFN-α2
IFN-γ
TNF-α
MCP-1
IL-12p70
IL-17A
IL-10
IL-18
IL-23
IL-33
IL-6
IL-8
PBS
LPS (100 ng/mL)
MiPro
MiPro + Sb++
Intensity
-1 0 1
Expression Level: Z-score
b
TNF-α (pg/mL)
Control
Sb+
Sb++
AMC
AMC + Sb+
AMC + Sb++
Van
Van + Sb+
Van + Sb++
c
Control
Sb++
AMC
AMC + Sb++

Figure S6

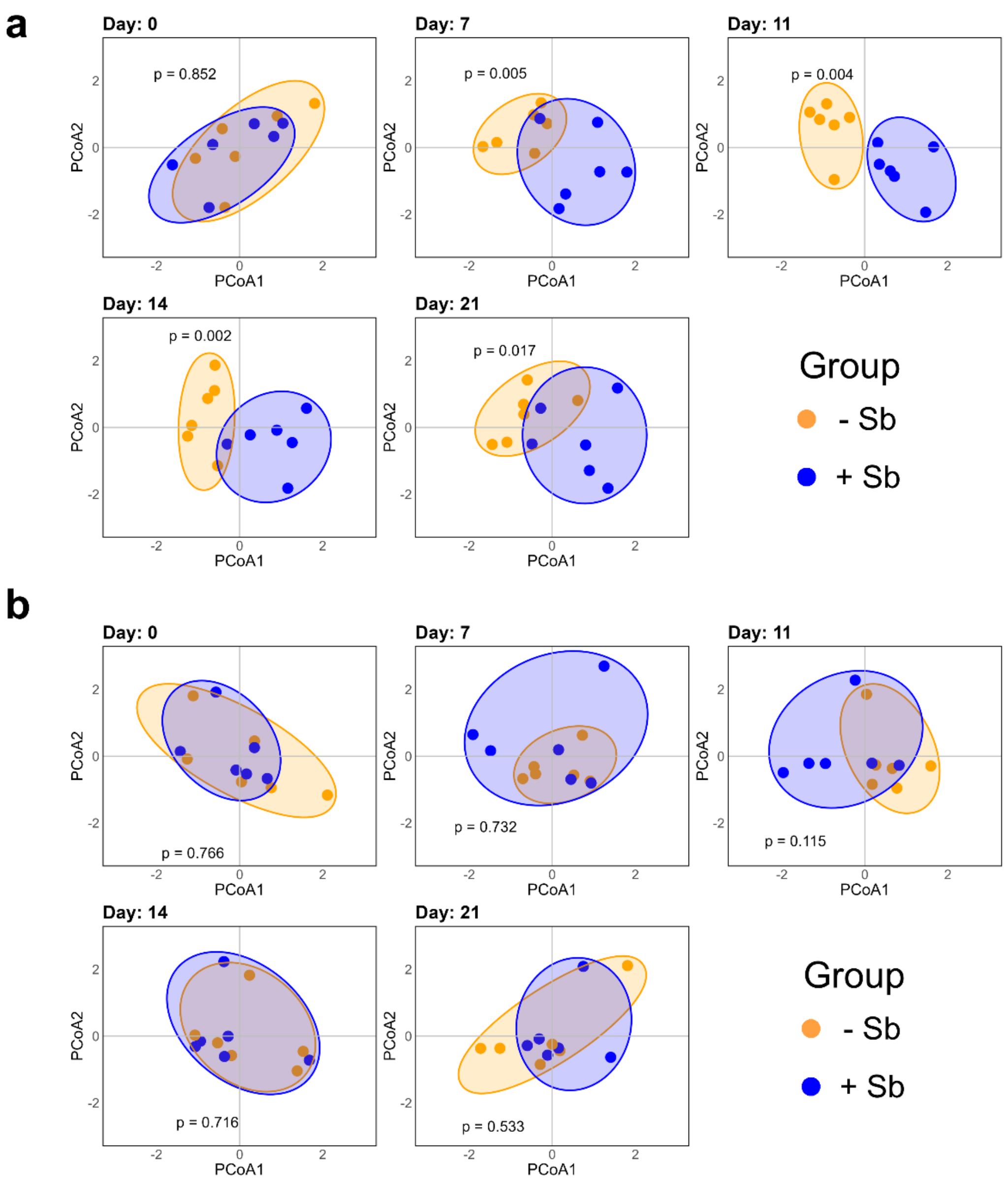
a
Day: 0
p = 0.852
Day: 7
p = 0.005
Day: 11
p = 0.004
Day: 14
p = 0.002
Day: 21
p = 0.017
PCoA1
PCoA2
Group
- Sb
+ Sb
b
Day: 0
p = 0.766
Day: 7
p = 0.732
Day: 11
p = 0.115
Day: 14
p = 0.716
Day: 21
p = 0.533
PCoA1
PCoA2
Group
- Sb
+ Sb

Figure S7

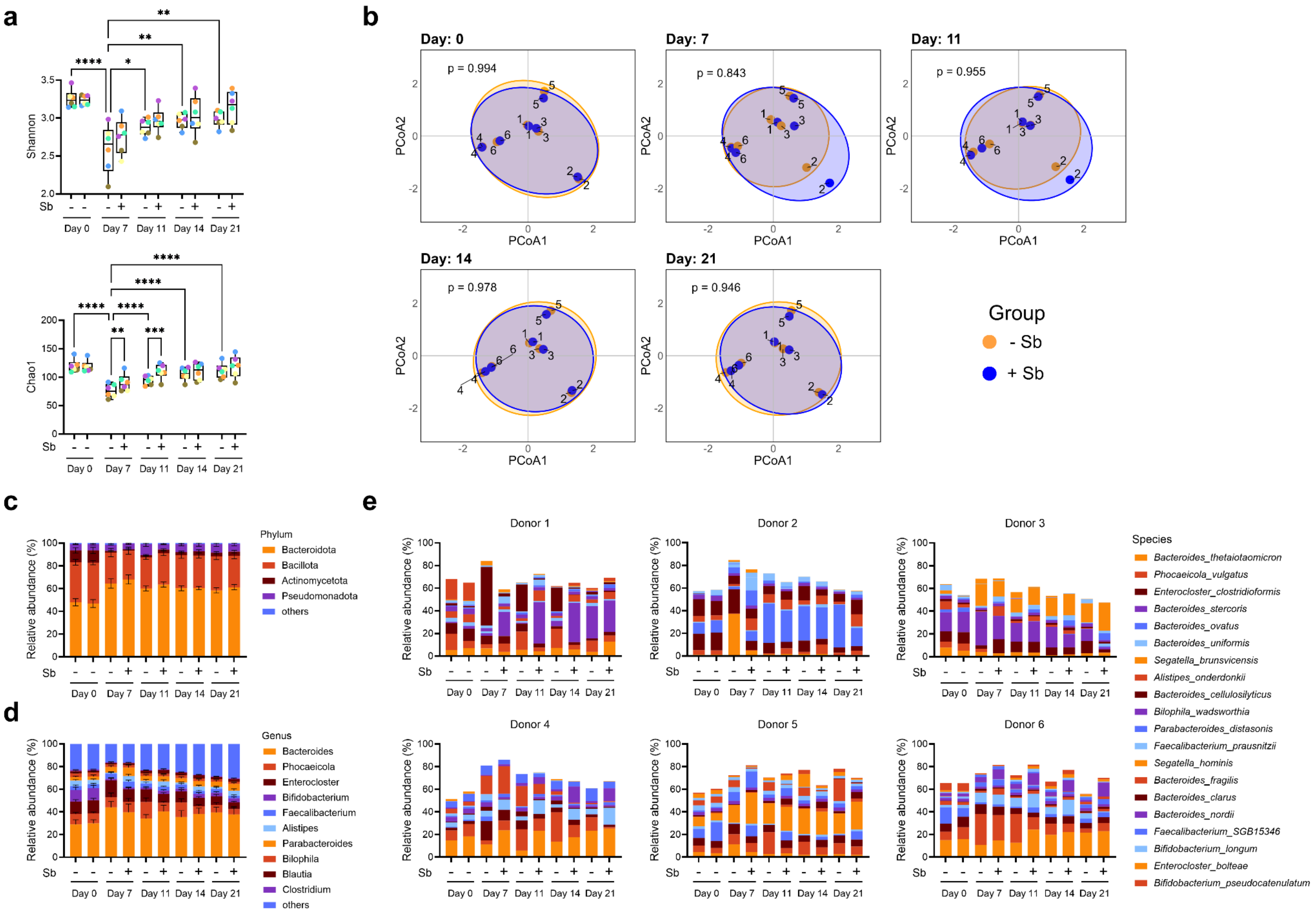
a
Shannon
Sb
Day 0
Day 7
Day 11
Day 14
Day 21
Chao1
b
Day: 0
p = 0.994
Day: 7
p = 0.843
Day: 11
p = 0.955
Day: 14
p = 0.978
Day: 21
p = 0.946
PCoA1
PCoA2
Group
- Sb
+ Sb
c
Relative abundance (%)
Phylum
Bacteroidota
Bacillota
Actinomycetota
Pseudomonadota
others
d
Genus
Bacteroides
Phocaeicola
Enterocloster
Bifidobacterium
Faecalibacterium
Alistipes
Parabacteroides
Bilophila
Blautia
Clostridium
others
e
Donor 1
Donor 2
Donor 3
Donor 4
Donor 5
Donor 6
Species
Bacteroides_thetaiotaomicron
Phocaeicola_vulgatus
Enterocloster_clostridioformis
Bacteroides_stercoris
Bacteroides_ovatus
Bacteroides_uniformis
Segatella_brunsvicensis
Alistipes_onderdonkii
Bacteroides_cellulosilyticus
Bilophila_wadsworthia
Parabacteroides_distasonis
Faecalibacterium_prausnitzii
Segatella_hominis
Bacteroides_fragilis
Bacteroides_clarus
Bacteroides_nordii
Faecalibacterium_SGB15346
Bifidobacterium_longum
Enterocloster_bolteae
Bifidobacterium_pseudocatenulatum

Figure S8

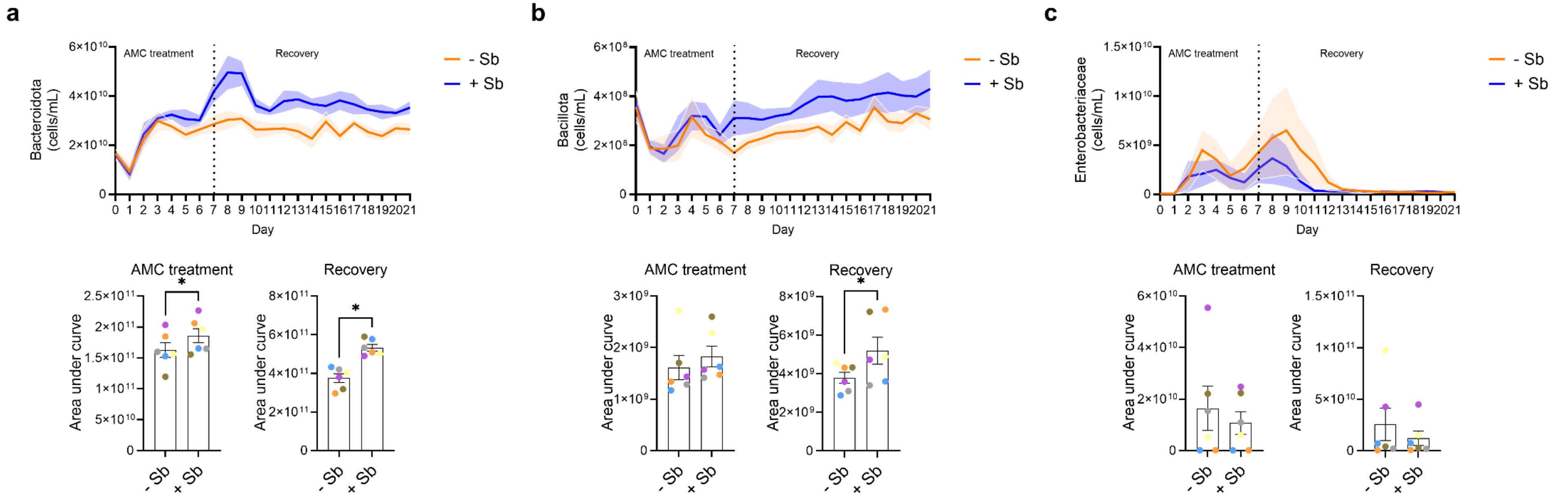

a
b
c
AMC treatment
Recovery
- Sb
+ Sb
Bacteroidota (cells/mL)
Bacillota (cells/mL)
Enterobacteriaceae (cells/mL)
Day
Area under curve
*

Figure S9

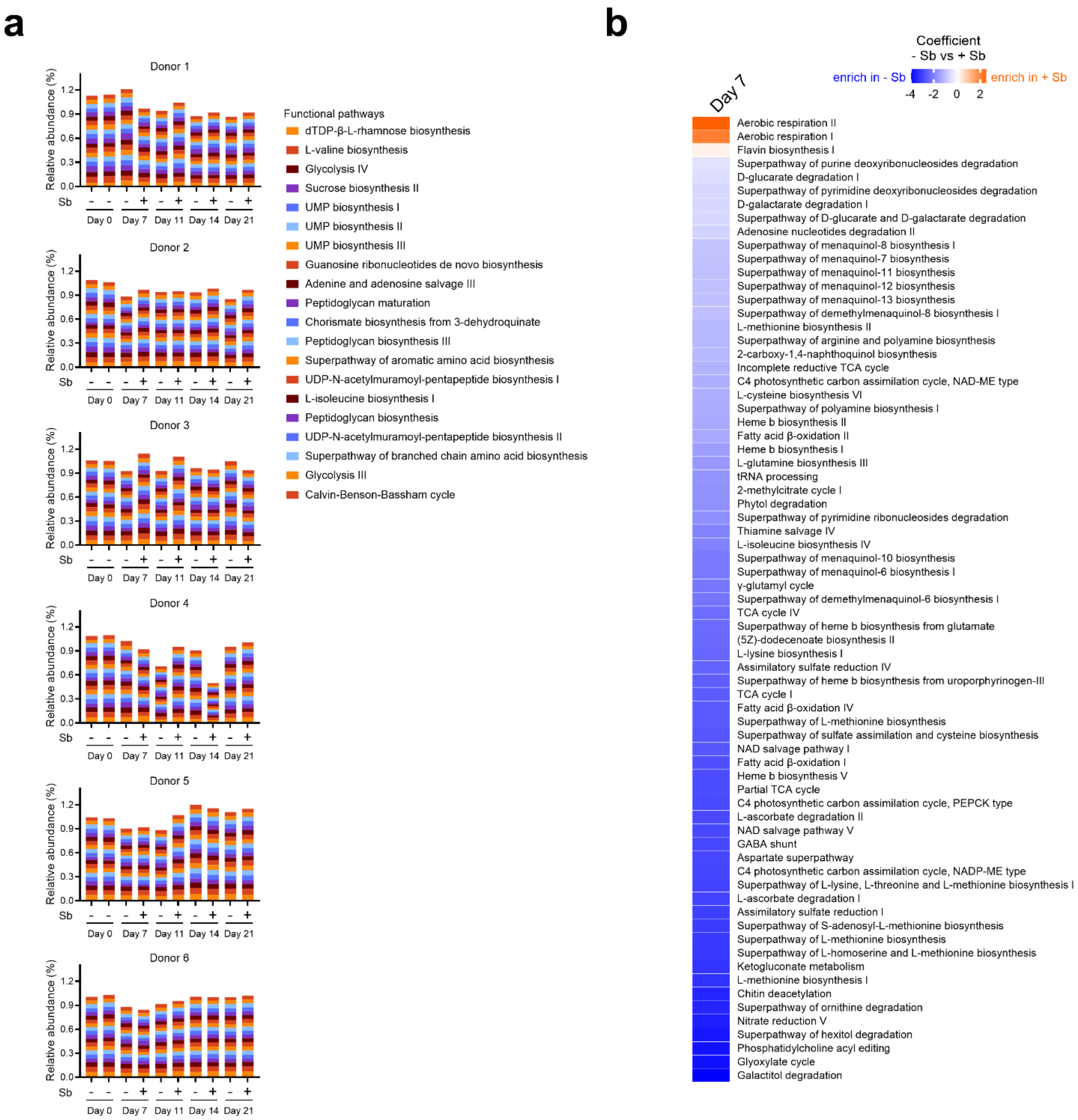
a
b
Donor 1
Donor 2
Donor 3
Donor 4
Donor 5
Donor 6
Relative abundance (%)
1.2
0.9
0.6
0.3
0.0
Sb
Day 0
Day 7
Day 11
Day 14
Day 21
Functional pathways
dTDP-β-L-rhamnose biosynthesis
L-valine biosynthesis
Glycolysis IV
Sucrose biosynthesis II
UMP biosynthesis I
UMP biosynthesis II
UMP biosynthesis III
Guanosine ribonucleotides de novo biosynthesis
Adenine and adenosine salvage III
Peptidoglycan maturation
Chorismate biosynthesis from 3-dehydroquinate
Peptidoglycan biosynthesis III
Superpathway of aromatic amino acid biosynthesis
UDP-N-acetylmuramoyl-pentapeptide biosynthesis I
L-isoleucine biosynthesis I
Peptidoglycan biosynthesis
UDP-N-acetylmuramoyl-pentapeptide biosynthesis II
Superpathway of branched chain amino acid biosynthesis
Glycolysis III
Calvin-Benson-Bassham cycle
Coefficient
- Sb vs + Sb
enrich in - Sb
enrich in + Sb
-4 -2 0 2
Day 7
Aerobic respiration II
Aerobic respiration I
Flavin biosynthesis I
Superpathway of purine deoxyribonucleosides degradation
D-glucarate degradation I
Superpathway of pyrimidine deoxyribonucleosides degradation
D-galactarate degradation I
Superpathway of D-glucarate and D-galactarate degradation
Adenosine nucleotides degradation II
Superpathway of menaquinol-8 biosynthesis I
Superpathway of menaquinol-7 biosynthesis
Superpathway of menaquinol-11 biosynthesis
Superpathway of menaquinol-12 biosynthesis
Superpathway of menaquinol-13 biosynthesis
Superpathway of demethylmenaquinol-8 biosynthesis I
L-methionine biosynthesis II
Superpathway of arginine and polyamine biosynthesis
2-carboxy-1,4-naphthoquinol biosynthesis
Incomplete reductive TCA cycle
C4 photosynthetic carbon assimilation cycle, NAD-ME type
L-cysteine biosynthesis VI
Superpathway of polyamine biosynthesis I
Heme b biosynthesis II
Fatty acid β-oxidation II
Heme b biosynthesis I
L-glutamine biosynthesis III
tRNA processing
2-methylcitrate cycle I
Phytol degradation
Superpathway of pyrimidine ribonucleosides degradation
Thiamine salvage IV
L-isoleucine biosynthesis IV
Superpathway of menaquinol-10 biosynthesis
Superpathway of menaquinol-6 biosynthesis I
γ-glutamyl cycle
Superpathway of demethylmenaquinol-6 biosynthesis I
TCA cycle IV
Superpathway of heme b biosynthesis from glutamate
(5Z)-dodecenoate biosynthesis II
L-lysine biosynthesis I
Assimilatory sulfate reduction IV
Superpathway of heme b biosynthesis from uroporphyrinogen-III
TCA cycle I
Fatty acid β-oxidation IV
Superpathway of L-methionine biosynthesis
Superpathway of sulfate assimilation and cysteine biosynthesis
NAD salvage pathway I
Fatty acid β-oxidation I
Heme b biosynthesis V
Partial TCA cycle
C4 photosynthetic carbon assimilation cycle, PEPCK type
L-ascorbate degradation II
NAD salvage pathway V
GABA shunt
Aspartate superpathway
C4 photosynthetic carbon assimilation cycle, NADP-ME type
Superpathway of L-lysine, L-threonine and L-methionine biosynthesis I
L-ascorbate degradation I
Assimilatory sulfate reduction I
Superpathway of S-adenosyl-L-methionine biosynthesis
Superpathway of L-methionine biosynthesis
Superpathway of L-homoserine and L-methionine biosynthesis
Ketogluconate metabolism
L-methionine biosynthesis I
Chitin deacetylation
Superpathway of ornithine degradation
Nitrate reduction V
Superpathway of hexitol degradation
Phosphatidylcholine acyl editing
Glyoxylate cycle
Galactitol degradation

Figure S10

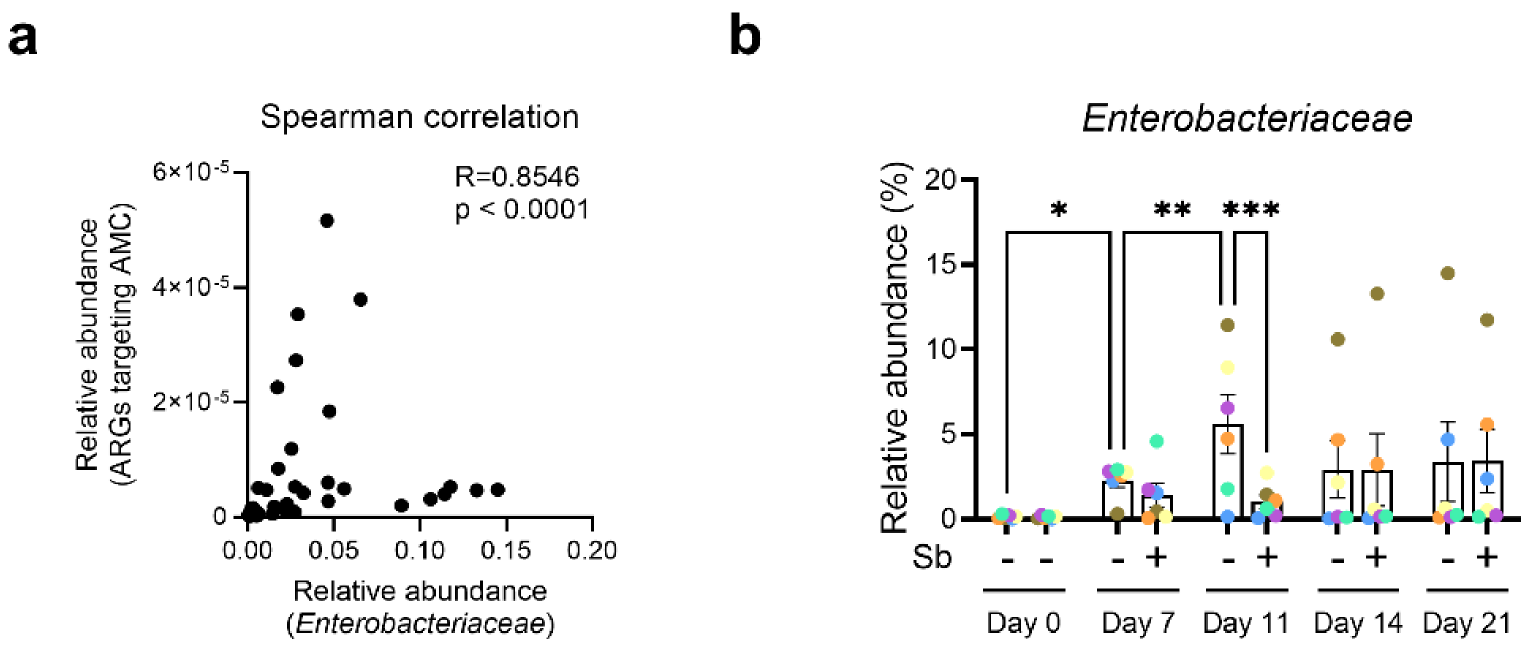
a
Spearman correlation
R=0.8546
p < 0.0001
Relative abundance (ARGs targeting AMC)
6×10-5
4×10-5
2×10-5
0
0.00 0.05 0.10 0.15 0.20
Relative abundance (Enterobacteriaceae)
b
Enterobacteriaceae
Relative abundance (%)
20
15
10
5
0
*
**
***
Sb
- - - + - + - + - +
Day 0
Day 7
Day 11
Day 14
Day 21

Figure S11

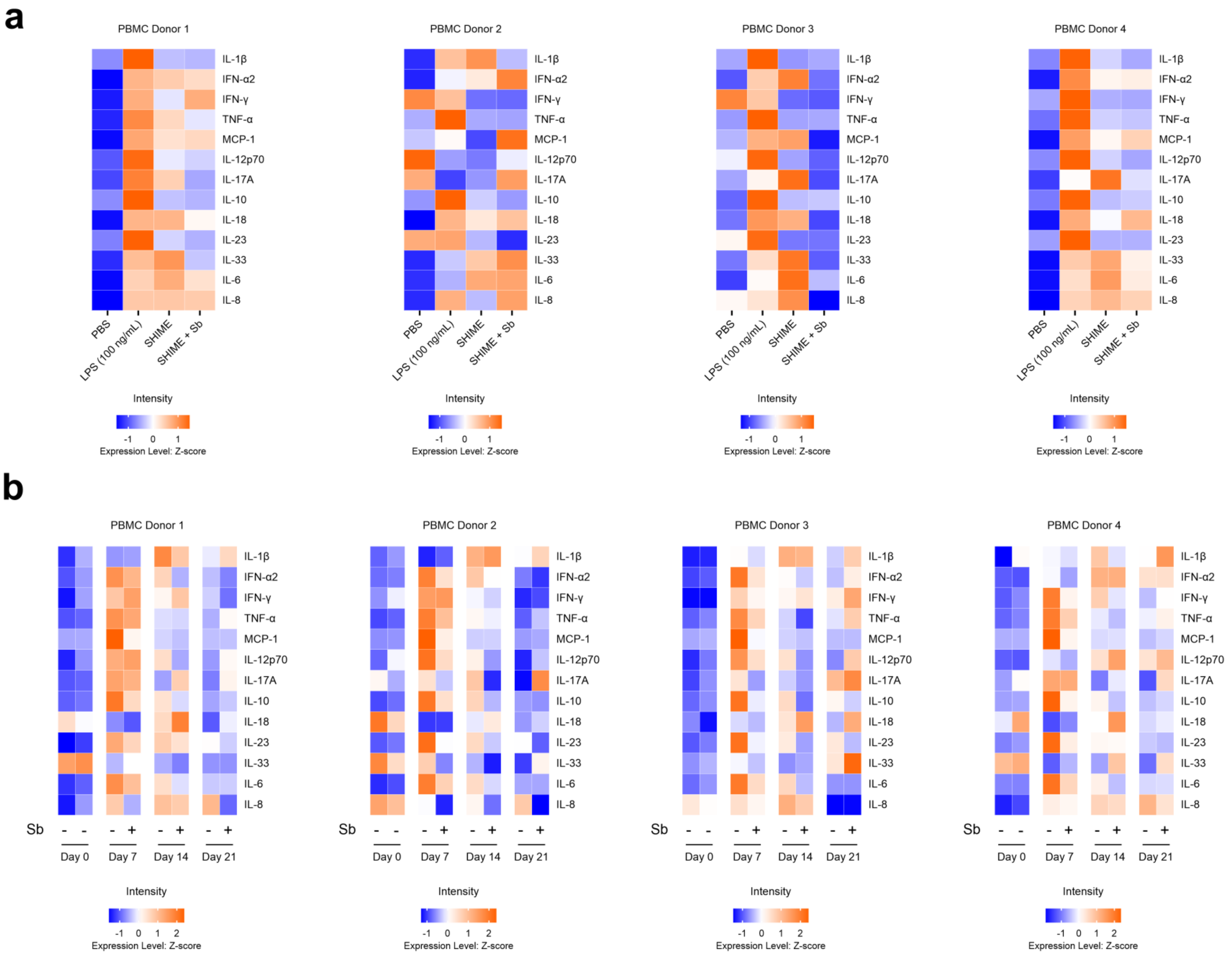
a
PBMC Donor 1
PBMC Donor 2
PBMC Donor 3
PBMC Donor 4
IL-1β
IFN-α2
IFN-γ
TNF-α
MCP-1
IL-12p70
IL-17A
IL-10
IL-18
IL-23
IL-33
IL-6
IL-8
PBS
LPS (100 ng/mL)
SHIME
SHIME + Sb
Intensity
-1 0 1
Expression Level: Z-score
b
Sb
- - - + - + - +
Day 0
Day 7
Day 14
Day 21
Intensity
-1 0 1 2
Expression Level: Z-score

Figure S12

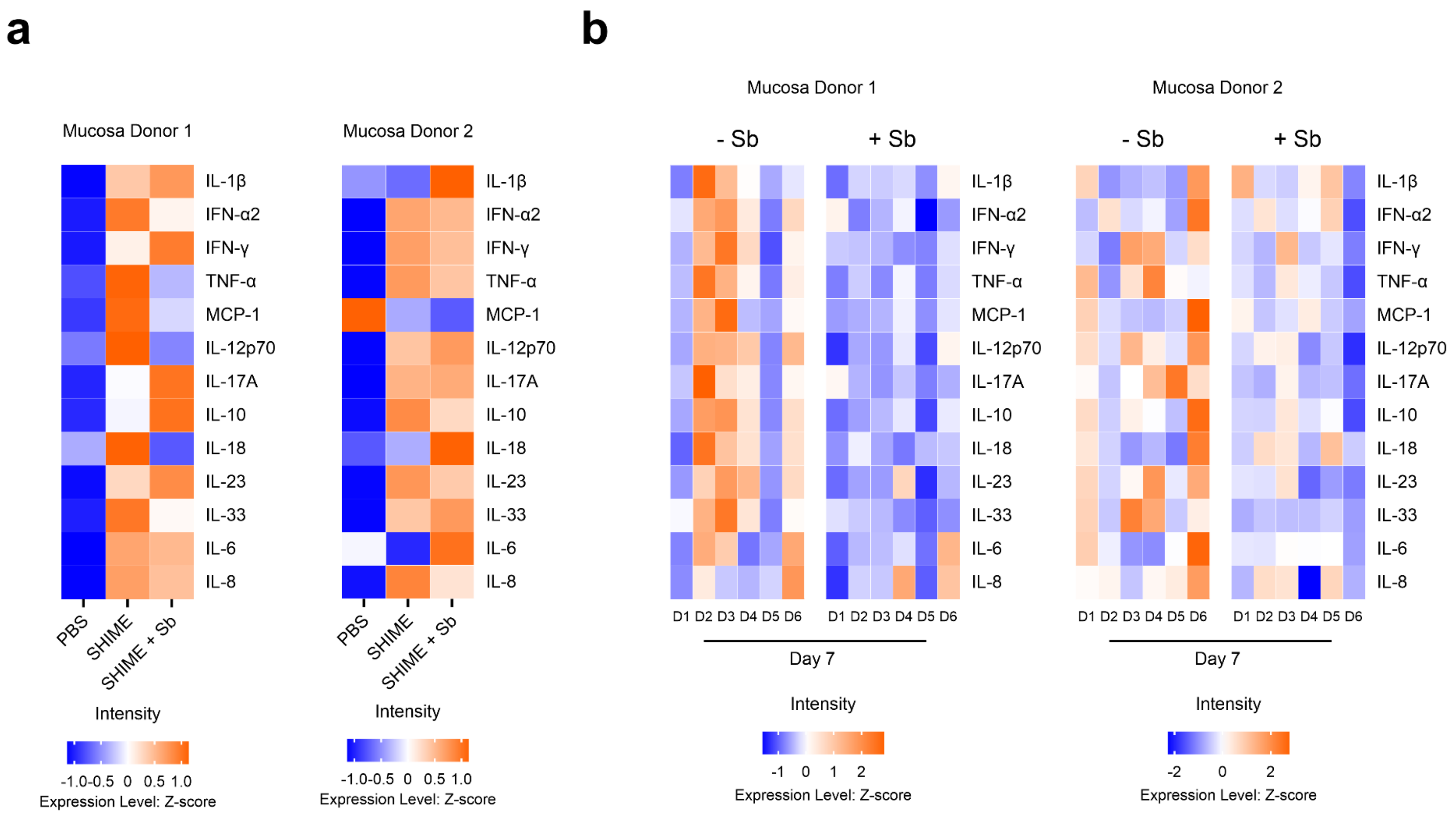
a
Mucosa Donor 1
Mucosa Donor 2
IL-1β
IFN-α2
IFN-γ
TNF-α
MCP-1
IL-12p70
IL-17A
IL-10
IL-18
IL-23
IL-33
IL-6
IL-8
PBS
SHIME
SHIME + Sb
Intensity
-1.0-0.5 0 0.5 1.0
Expression Level: Z-score
b
Mucosa Donor 1
Mucosa Donor 2
- Sb
+ Sb
D1 D2 D3 D4 D5 D6
Day 7
Intensity
-1 0 1 2
-2 0 2
Expression Level: Z-score

Figure S13
